# Development of a Nomogram Based on 3D CT Radiomics Signature to Predict the Mutation Status of EGFR Molecular Subtypes in Lung Adenocarcinoma: A Multicenter Study

**DOI:** 10.3389/fonc.2022.889293

**Published:** 2022-04-29

**Authors:** Guojin Zhang, Liangna Deng, Jing Zhang, Yuntai Cao, Shenglin Li, Jialiang Ren, Rong Qian, Shengkun Peng, Xiaodi Zhang, Junlin Zhou, Zhuoli Zhang, Weifang Kong, Hong Pu

**Affiliations:** ^1^Department of Radiology, Sichuan Provincial People’s Hospital, University of Electronic Science and Technology of China, Chengdu, China; ^2^Department of Radiology, Chinese Academy of Sciences Sichuan Translational Medicine Research Hospital, Chengdu, China; ^3^Department of Radiology, Lanzhou University Second Hospital, Lanzhou, China; ^4^Department of Radiology, Fifth Affiliated Hospital of Zunyi Medical University, Zhuhai, China; ^5^Department of Radiology, Affiliated Hospital of Qinghai University, Xining, China; ^6^Department of Pharmaceuticals Diagnosis, GE Healthcare, Beijing, China; ^7^Clinical Science Department, Philips (China) Investment Co., Ltd., Chengdu, China; ^8^Department of Radiology and BME, University of California Irvine, Irvine, CA, United States

**Keywords:** NSCLC, lung adenocarcinoma, EGFR, computed tomography, radiomics

## Abstract

**Background:**

This study aimed to noninvasively predict the mutation status of epidermal growth factor receptor (EGFR) molecular subtype in lung adenocarcinoma based on CT radiomics features.

**Methods:**

In total, 728 patients with lung adenocarcinoma were included, and divided into three groups according to EGFR mutation subtypes. 1727 radiomics features were extracted from the three-dimensional images of each patient. Wilcoxon test, least absolute shrinkage and selection operator regression, and multiple logistic regression were used for feature selection. ROC curve was used to evaluate the predictive performance of the model. Nomogram was constructed by combining radiomics features and clinical risk factors. Calibration curve was used to evaluate the goodness of fit of the model. Decision curve analysis was used to evaluate the clinical applicability of the model.

**Results:**

There were three, two, and one clinical factor and fourteen, thirteen, and four radiomics features, respectively, which were significantly related to each EGFR molecular subtype. Compared with the clinical and radiomics models, the combined model had the highest predictive performance in predicting EGFR molecular subtypes [Del-19 mutation *vs.* wild-type, AUC=0.838 (95% CI, 0.799-0.877); L858R mutation *vs.* wild-type, AUC=0.855 (95% CI, 0.817-0.894); and Del-19 mutation *vs.* L858R mutation, AUC=0.906 (95% CI, 0.869-0.943), respectively], and it has a stable performance in the validation set [AUC was 0.813 (95% CI, 0.740-0.886), 0.852 (95% CI, 0.790-0.913), and 0.875 (95% CI, 0.781-0.929), respectively].

**Conclusion:**

Our combined model showed good performance in predicting EGFR molecular subtypes in patients with lung adenocarcinoma. This model can be applied to patients with lung adenocarcinoma.

## Introduction

Targeted therapy has brought based on recognizing the importance of acquired gene driver mutations, such as epidermal growth factor receptor (EGFR) mutations, kristen rat sarcoma (KRAS) mutations and anaplastic lymphoma kinase (ALK) rearrangements, in non-small cell lung cancer (NSCLC) new hope to patients with these gene mutations. In the Asian population, about 50% of lung adenocarcinoma patients have known carcinogenic driver genes (1, 2). There are currently targeted drugs used in clinical practice for these mutations, such as gefitinib and osimertinib for EGFR mutations. In contrast, patients without these mutations are not candidates for targeted therapy (3). Furthermore, there are molecular differences between each molecular mutation and molecular subtype, and these differences lead to different therapeutic effects after using other targeted drugs (4). EGFR mutations mainly include exon 18-21 mutations. Among them, exon 19 deletion (Del-19) mutation and 21 L858R point (L858R) mutation are the two most common activating mutations, and they are also the two most sensitive mutation sites for tyrosine kinase inhibitors (TKI) treatment (5). In a single targeted therapy, patients with Del-19 mutation benefited more from osimertinib (6), while patients with L858R mutation benefited significantly from dacomitinib (7); in addition, combination therapy and immunotherapy brought patients with L858R for more potential benefits (8, 9). Therefore, the detection of specific EGFR mutation subtypes can make targeted therapies more precise and allow patients receiving these treatments to benefit the most.

Currently, the detection of EGFR mutation status from histological specimens is the most common detection method. However, in clinical practice, these detection techniques also have some limitations. For example, tissue samples are obtained through invasive methods such as biopsy or surgery; sometimes the amount of tissue samples obtained due to operational errors is insufficient; biopsy can increase the risk of tumor metastasis; in addition, a small part of the tissue obtained does not represent the heterogeneity of the entire tumor, etc. (10–12). In addition, another noninvasive detection strategy for EGFR mutations is ‘liquid biopsy’, which is a biological detection method on the blood. For patients with advanced NSCLC, ‘liquid biopsy’ is a promising method to isolate circulating tumor DNA from blood samples (13). However, ‘liquid biopsy’ has a high risk of false-negative results (30%) (14). Therefore, until this defect is effectively resolved, ‘liquid biopsy’ is far from substitute for histological testing. Because of this, there is an urgent need for a simple and noninvasive method to detect EGFR mutation subtypes before targeted drug therapy.

The radiological features have been shown to reflect EGFR mutation status in lung adenocarcinoma (12, 15–17). However, the clinical applicability of these studies needs to be confirmed by further research. Compared with traditional CT, radiomics converts medical images into mineable data and extracts a large number of features that cannot be observed by the human naked eye system, thereby reflecting more characteristics of tumors (18). To our knowledge, some studies have used radiomics to predict EGFR mutation status (19–22). Although the prediction performance of these studies is different, this shows that it is feasible to predict EGFR mutations noninvasively through radiomics. However, only a few studies have used radiomics methods to predict the mutation status of EGFR molecular subtypes (23–26). Unfortunately, the sample size included in these studies is limited, and the accuracy of the obtained prediction model was only 65.5-79.0%. In this study, we retrospectively collected a relatively large data set and constructed a model based on CT radiomics signature to noninvasively predict the mutation status of EGFR molecular subtype in lung adenocarcinoma.

## Materials and Methods

### Patient Population

This retrospective study was ethically approved by the Institutional Review Board of the Sichuan Provincial People’s Hospital and Lanzhou University Second Hospital, and the need for patient informed consent was waived. Clinical data and chest CT images of these patients were obtained from the picture archiving and communication system (PACS). The inclusion criteria were as follows: (1) patients with the histologic type of lung adenocarcinoma; (2) patients with complete CT thin-slice images (1.25 mm) and clinical data; (3) patients who did not receive lung cancer-related treatment before CT scan; (4) patients who underwent biopsy or surgery within one month after CT scan; (5) patients with EGFR exon Del-19 mutation, exon L858R mutation, and wild-type. The exclusion criteria were as follows: (1) patients whose tumor boundary is difficult to be recognized by the naked eye on CT images; (2) patients younger than 18 years old.

**Figure 1:**
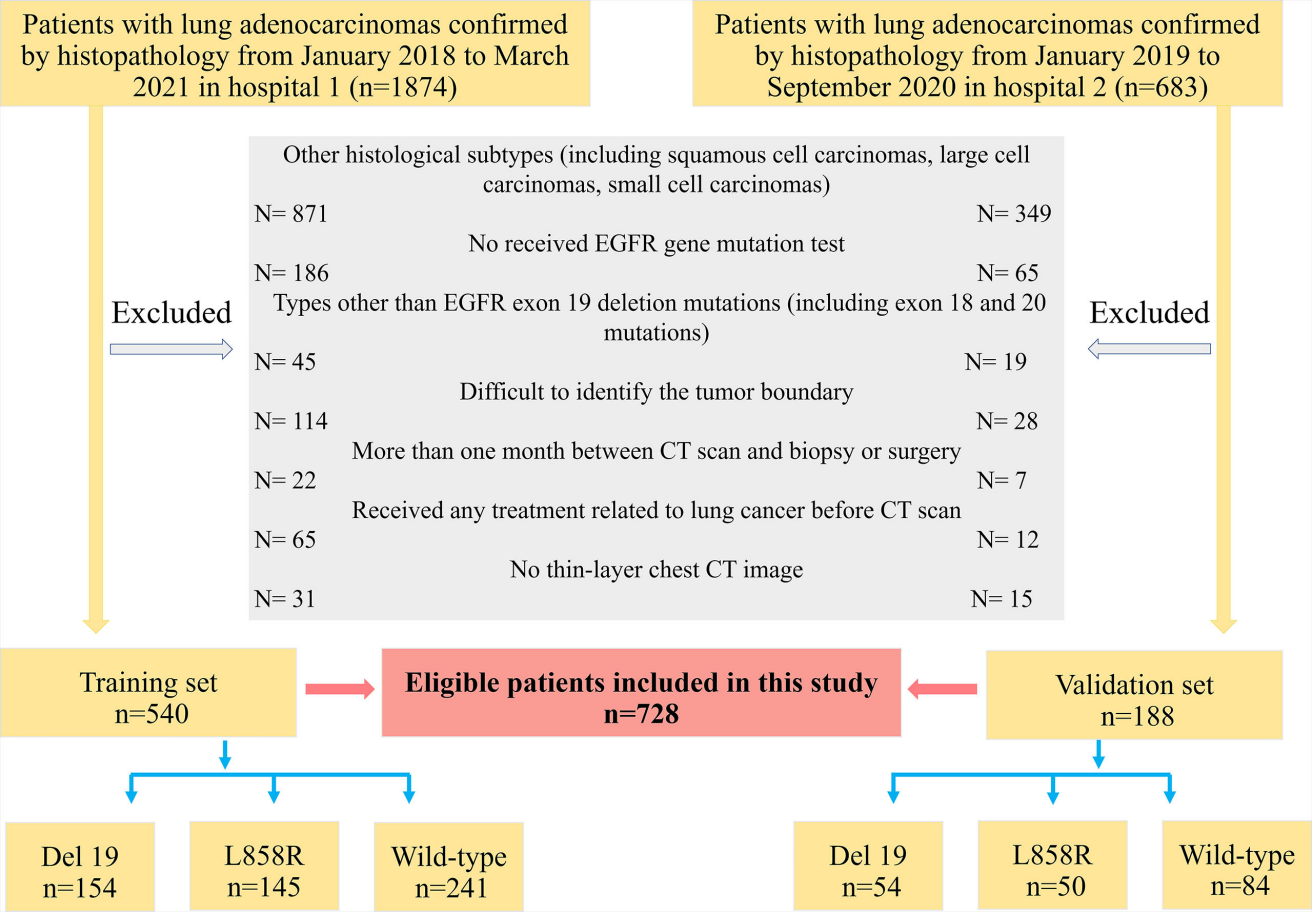
The flowchart of the inclusion and exclusion criteria.

According to the above inclusion and exclusion criteria, 728 patients (median age, 57.0 years, age range, 21-82 years, 370 males and 358 females) were finally selected from 2,557 patients in the two hospitals. Among them, a total of 540 patients from Sichuan Provincial People’s Hospital were used as the training set from January 2018 to March 2021, and 188 patients from Lanzhou University Second Hospital were identified as the external validation set from January 2019 to September 2020. The patient recruitment flowchart is shown in **Figure 1**.

Demographic and clinical data include the patient’s sex, age, smoking history [including non-smoking (never smoked) and smoking (former or current smoking)], carcinoembryonic antigen (CEA) level, and tumor lobe location of the tumor (including right upper, right middle, right lower, left upper and left lower lobes). If the tumor crosses the fissure, the lobe location is defined as the lobe in which the tumor predominates.

### EGFR Mutation Status Detection

The polymerase chain reaction-amplified refractory mutation system (PCR-ARMS) detected EGFR mutation status. The human EGFR gene detection kit (Beijing SinoMD Gene Detection Technology Co., Ltd., China; Amoy Diagnostics, Xiamen, China) detected EGFR exon 18 to 21 mutation status.

### CT Image Acquisition

CT scans ranged from the thoracic inlet to the level of the lower edge of the 12th rib were completed by three spiral CT scanners (Discovery CT750 HD, GE Healthcare; Philips iCT 256, Koninklijke Philips N.V.; Somatom Sensation 64, Siemens Healthineers). Scanning parameters were as follows: (1) tube voltage 120 kVp, tube current adjusted automatically for the Sensation 64 scanner, and (2) tube voltage 120 kVp, tube current 150 to 200 mA for the other two scanners. For all scanners, 0.5-1.0 second tube rotation time, and field of view (FOV): 350 mm; matrix, 512 × 512; the layer thickness and spacing were both 5 mm; the reconstruction layer thickness and spacing were both 1.25 mm. All images were exported in DICOM format to facilitate feature extraction.

### Tumor Segmentation and Radiomics Feature Extraction

**Figure 2:**
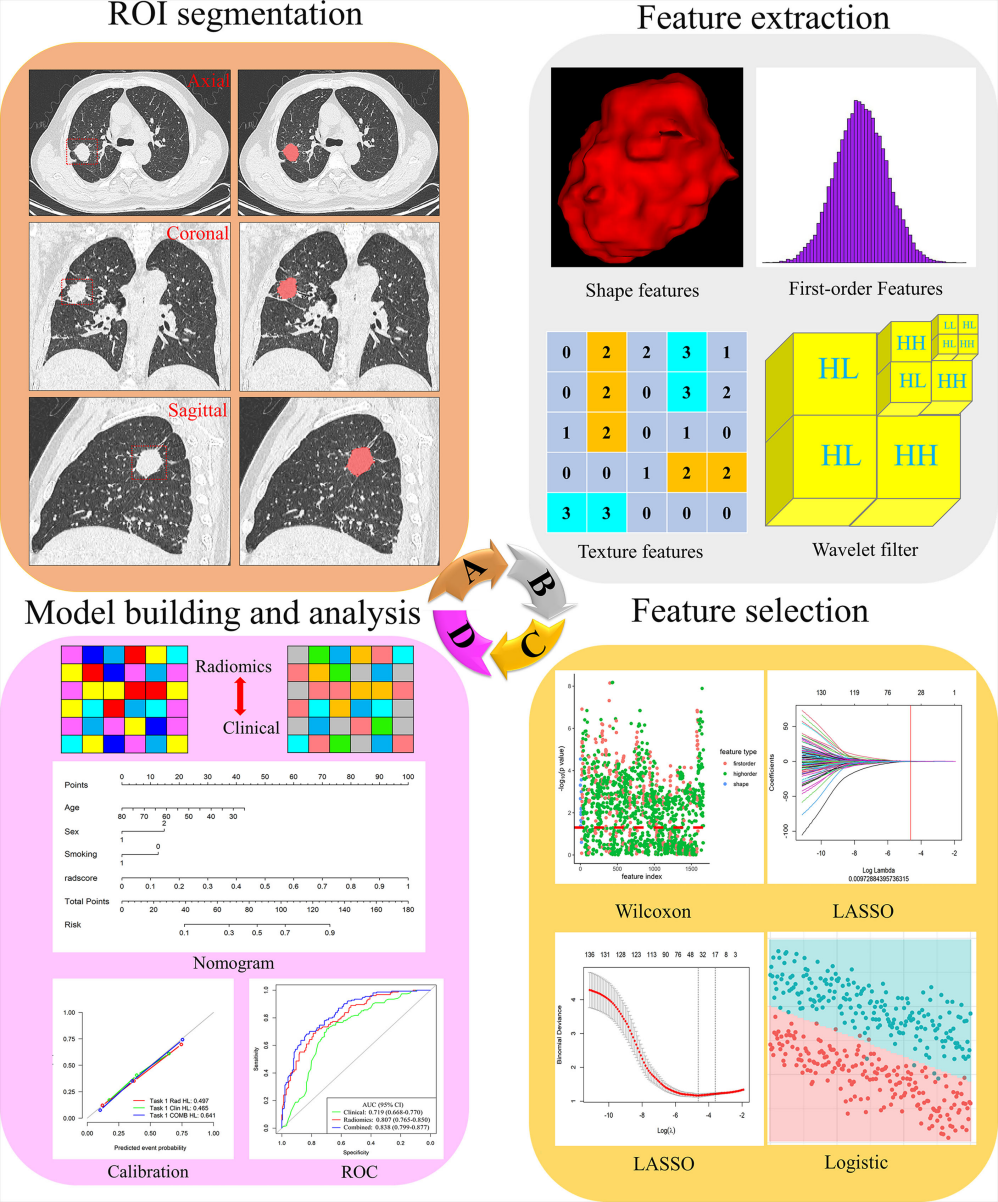
Flowchart of the process of radiomics. **(A)** The tumours were segmented on CT images to form the region of interest (ROI). **(B)** Radiomics feature extraction from the ROI. **(C)** Radiomics feature dimensionality reduction process. **(D)** Construct a radiomics model.

Radiomics feature extraction and analysis workflow are shown in **Figure 2**. To ensure the accuracy and consistency of the data, two readers (radiologists with 6 and 4 years of experience in chest CT diagnosis, respectively) independently used the open-source software ITK-SNAP 3.8.0 (http://www.itksnap.org) to segment the tumor on the thin-slice CT lung window (window width: 1500HU; window level: -500HU). Training cases were segmented by reader 1 (G.J.Z), and validation cases were segmented by reader 2 (L.N.D). Both readers were blinded to all patients’ clinical data, pathological records, and EGFR status results. When the two readers were unsure, a consultant radiologist (J.Z) confirmed the segmentation with 17 years of experience. The region of interest (ROI) was manually segmented on CT axial images with tumor tissue and confirmed on sagittal and coronal images.

In order to evaluate the robustness and repeatability of the radiomics feature extraction process, one month later, 40 patients were randomly selected from the training set and segmented again by readers 1 and 2 to construct a re-segmentation set, and 40 patients were randomly selected from each CT scanner to construct different CT scanner sets for calculating the intra-/interclass correlation coefficients (ICC), respectively. ICC values > 0.8 reflected good consistency (26).

The open-source Python software package PyRadiomics 3.0.1 automatically extracted radiomics features from the three-dimensional (3D) tumor volume after segmentation. Radiomics features were divided into three main categories: 16 shape features, 324 first-order features and 1387 texture features. Details of radiomics features were included in the **Supplementary Material** (Methods). According to the recommendations of International Symposium on Biomedical Imaging (ISBI), we only resampled the image and set the bin width of gray discretization to 25. We performed z-score preprocessing on the extracted radiomics features.

### Radiomics Feature Selection

To avoid model overfitting and improve accuracy, we used three steps for feature selection to obtain the optimal feature subset. First, Wilcoxon test was used to retain the features with *P*-value less than 0.05. Secondly, the least absolute shrinkage and selection operator (LASSO) regression with 10-fold cross-validation was used to eliminate the collinearity features. LASSO is a recognized algorithm that has been used for feature selection of higher-dimensional variables (27). Finally, multiple logistic regression was used to select the features, and the minimum features of akaike information criterion (AIC) were retained.

For clinical factors, the Chi-square and Student’s *t*-tests were first used in the training set to screen for clinical characteristics that were correlated between each group. *P*-value was set to 0.05. Clinical factors with a *P*-value greater than 0.05 were excluded. Next, use logistic regression to further analyze the most relevant variables.

### Radiomics Model Establishment

Logistic regression was used in the training set to build a model for predicting Del-19 or L858R mutations, and its performance was evaluated in the external validation set. Logistic regression is a classic method in radiomics research. It is easy to understand, explain, and combine discrete and continuous variables (28, 29). To identify Del 19 and L858R mutations, we used logistic regression, support vector machine (SVM), and random forest (RF) to construct prediction models. The clinical and radiomics models were constructed based on clinical factors and radiomics features, respectively, while the combined model was constructed based on clinical and radiomics models. Additionally, clinical models were constructed using logistic regression.

### Statistical analysis

All statistical analyses were performed using R 3.6.0 (http://www.R-project.org). Two-sided *P*-values less than 0.05 were considered to be statistically different. Kolmogorov-Smirnov test was used to evaluate the normal distribution of the data. Categorical variables were expressed as percentiles, and the Chi-square test or Fisher’s exact test was used to analyze groups. Continuous variables were expressed as mean ± standard deviation (SD), and Student’s *t*-test or Mann-Whitney U test was used for analysis between groups. Receiver operating characteristic (ROC) curve was used to evaluate the performance of the model, and the area under the curve (AUC), sensitivity, specificity, accuracy, positive predictive value (PPV), and negative predictive value (NPV) were calculated. Delong test was used to compare the performance differences of the prediction models.

Based on the above-screened clinical factors and radiomics features, we constructed a personalized nomogram to predict the mutation status of the EGFR molecular subtype. Calibration curve and Hosmer-Lemeshow (H-L) test were used to evaluate the model’s goodness of fit. Decision curve analysis (DCA) was used to assess the clinical applicability of the model.

## Results

### Clinical Characteristics of Patients

There were no significant differences in clinical factors (including sex, smoking history, and CEA), the mutation rate of Del-19 or L858R, and tumor location in each EGFR mutant subtype group (all *P* > 0.05), while there were significant differences in age between the two EGFR mutant subtype groups (Del-19 *vs.* wild type, Del-19 *vs.* L858R) (**Supplementary Tables S1–S3**).

Univariate analysis revealed that age, sex and smoking history were significantly different between the Del-19 mutation and wild-type groups (*P* < 0.05), sex and smoking history were significantly different between the L858R mutation and wild-type groups (*P* < 0.05), and age was significantly different between the Del-19 mutation and L858R mutation groups (*P* < 0.05). Multivariate analysis revealed that age (OR, 0.972; 95% CI, 0.948-0.996; *P* = 0.021) and sex (OR, 3.193; 95% CI, 1.836-5.565; *P* < 0.001) were correlated independently with the task of Del-19 *vs.* wild-type (**Table 1**), sex (OR, 2.612; 95% CI, 1.548-4.457; *P* < 0.001) and smoking history (OR, 0.427; 95% CI, 0.238-0.761; *P* = 0.001) were correlated independently with the task of L858R *vs.* wild-type (**Table 2**), and age (OR, 1.050; 95% CI, 1.022-1.081; *P* < 0.001) was correlated independently with the task of Del-19 *vs.* L858R (**Table 3**). Based on multivariate analysis results, clinical factors with *P* < 0.05 in each task were incorporated in the clinical model.

**Table 1:** The relationship between clinical variables of patients and EGFR molecular subtypes (Del-19 mutation *vs.* Wild-type) in the training set.

Variable	Total (n = 395)	Del-19 mutation (n =154)	Wild-type (n = 241)	`Univariate analysis	Multivariate analysis		
				*P* value	OR (95%CI)	*P* value	
Age (years)				<0.001	0.972 (0.948-0.996)		0.021
- Mean ± SD	56.70 ± 9.19	54.87 ± 8.13	58.87 ± 9.65				
- Median (Q_1_, Q_3_)	56.0 (50.0,63.0)	55.0(49.0,61.0)	58.0(51.0,65.0)				
- Range	26-79	32-78	26-79				
Sex (%)				<0.001			<0.001
- Male	221 (55.9%)	51 (33.1%)	170 (70.5%)		Reference		
- Female	174 (44.1%)	103 (66.9%)	71 (29.5%)		3.193 (1.836-5.656)		
Smoking history (%)				<0.001	NA		
- No	240 (60.8%)	122 (79.2%)	118 (49.0%)				
- Yes	155 (39.2%)	32 (20.8%)	123 (51.0%)				
CEA (%)				0.391	NA		
- Normal	167 (42.3%)	61 (39.6%)	106 (44.0%)				
- High	228 (57.7%)	93 (60.4%)	135 (56.0%)				
Lobe location (%)				0.959	NA		
- Right upper lobe	135 (34.2%)	54 (35.1%)	81 (33.6%)				
- Right middle lobe	17 (4.3%)	6 (3.9%)	11 (4.6%)				
- Right lower lobe	97 (24.6%)	35 (22.7%)	62 (25.7%)				
- Left upper lobe	81 (20.5%)	33 (21.4%)	48 (19.9%)				
- Left lower lobe	65 (16.5%)	26 (16.9%)	39 (16.2%)				

CEA, Carcinoembryonic antigen; CI, Confidence interval; Del 19, Exon-19 deletion mutation; EGFR, Epidermal growth factor receptor; NA, not applicable; OR, Odds ratio; SD, Standard deviation. vs., versus.

**Table 2:** The relationship between clinical variables of patients and EGFR molecular subtypes (L858R mutation *vs.* Wild-type) in the training set.

Variable	Total (n = 386)	L858R mutation (n = 145)	Wild-type (n = 241)	Univariate analysis	Multivariate analysis	
				*P* value	OR (95%CI)	*P* value
Age (years)				0.686	NA	
- Mean ± SD	58.12 ± 9.46	58.50 ± 9.15	58.87 ± 9.65			
- Median (Q_1_, Q_3_)	58.0 (52.0,65.0)	58.0(53.0,64.0)	58.0(51.0,65.0)			
- Range	21-82	21-82	26-79			
Sex (%)				<0.001		<0.001
- Male	223 (57.8%)	53 (36.6%)	170 (70.5%)		Reference	
- Female	174 (44.2%)	92 (63.4%)	71 (29.5%)		2.612 (1.548-4.457)	
Smoking history (%)				<0.001		0.004
- No	234 (60.6%)	116 (80.0%)	118 (49.0%)		Reference	
- Yes	152 (39.4%)	29 (20.0%)	123 (51.0%)		0.427 (0.238-0.761)	
CEA (%)				0.301	NA	
- Normal	162 (42.0%)	56 (38.6%)	106 (44.0%)			
- High	224 (58.0%)	89 (61.4%)	135 (56.0%)			
Lobe location (%)				0.262	NA	
- Right upper lobe	124 (32.1%)	43 (29.7%)	81 (33.6%)			
- Right middle lobe	24 (6.2%)	13 (9.0%)	11 (4.6%)			
- Right lower lobe	99 (25.6%)	37 (25.5%)	62 (25.7%)			
- Left upper lobe	83 (21.5%)	35 (24.1%)	48 (19.9%)			
- Left lower lobe	56 (14.5%)	17 (11.7%)	39 (16.2%)			

CEA, Carcinoembryonic antigen; CI, Confidence interval; EGFR, Epidermal growth factor receptor; L858R, Exon-21 L858R point mutation; NA, not applicable; OR, Odds ratio; SD, Standard deviation. vs., versus.

**Table 3:** The relationship between clinical variables of patients and EGFR molecular subtypes (Del-19 mutation *vs.* L858R mutation) in the training set.

Variable	Total (n = 299)	Del-19 mutation (n = 154)	L858R mutation (n = 145)	Univariate analysis	Multivariate analysis	
				*P* value	OR (95%CI)	*P* value
Age (years)				<0.001	1.050 (1.022-1.081)	<0.001
- Mean ± SD	56.63 ± 8.81	54.87 ± 8.13	58.50 ± 9.15			
- Median (Q_1_, Q_3_)	56.0 (50.5,62.5)	55.0(49.0,61.0)	58.0(53.0,64.0)			
- Range	21-82	32-78	21-82			
Sex (%)				0.533	NA	
- Male	104 (34.8%)	51 (33.1%)	53 (36.6%)			
- Female	195 (65.2%)	103 (66.9%)	92 (63.4%)			
Smoking history (%)				0.867	NA	
- No	238 (79.6%)	122 (79.2%)	116 (80.0%)			
- Yes	61 (20.4%)	32 (20.8%)	29 (20.0%)			
CEA (%)				0.861	NA	
- Normal	117 (39.1%)	61 (39.6%)	56 (38.6%)			
- High	182 (60.9%)	93 (60.4%)	89 (61.4%)			
Lobe location (%)				0.235	NA	
- Right upper lobe	97 (32.4%)	54 (35.1%)	43 (29.7%)			
- Right middle lobe	19 (6.4%)	6 (3.9%)	13 (9.0%)			
- Right lower lobe	72 (24.1%)	35 (22.7%)	37 (25.5%)			
- Left upper lobe	68 (22.7%)	33 (21.4%)	35 (24.1%)			
- Left lower lobe	43 (14.4%)	26 (16.9%)	17 (11.7%)			

CEA, Carcinoembryonic antigen; CI, Confidence interval; Del 19, Exon-19 deletion; EGFR, Epidermal growth factor receptor; L858R, Exon-21 L858R point mutation; NA, not applicable; OR, Odds ratio; SD, Standard deviation. vs., versus.

### Radiomics Feature Selection and Model Establishment

In total, 1727 radiomics features were extracted from the 3D images of each ROI. The ICC values of the radiomics features extracted from two readers and different CT scanners were all greater than 0.80, reflecting good consistency. Fourteen radiomics features were highly correlated with Del-19 mutation (**Table S4; Figure S1**), thirteen radiomics features were highly correlated with L858R mutation (**Table S4; Figure S2**). For Del-19 mutation *vs.* L858R mutation, only four radiomics features were screened after using the Wilcoxon test (**Table S4; Figure S3**). Therefore, we retained these four features to construct the prediction model.

Correlation analysis showed that the correlation between each feature is weak and independent in the training and validation sets (**Figures S4–S6**).

Based on the above-screened radiomics features and clinical factors, the clinical, radiomics, and combined models were established in the training set, respectively, to predict the EGFR molecular subtype mutation status.

### Predictive performance and Validation Based on Clinical, Radiomics, and Combined Models

**Figure 3:**
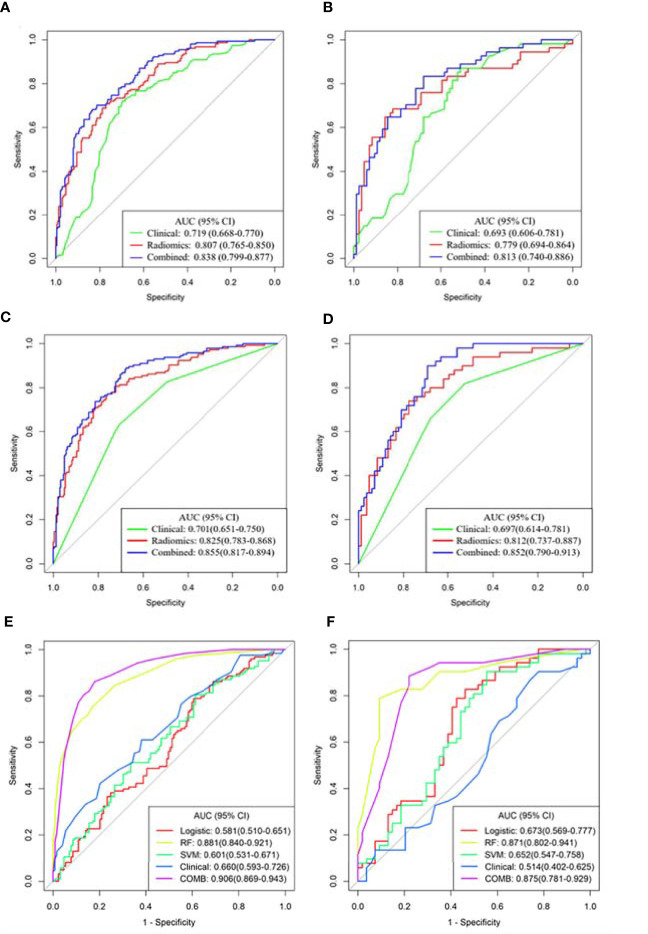
Receiver operating characteristic (ROC) curves of the three models were used to predict the mutant status of EGFR molecular subtypes. **(A, B)** Del-19 mutation *vs*. wild-type. **(C, D)** L858R mutation *vs.* wild-type. **(E, F)** Del-19 mutation *vs.* L858R mutation. **(A, C, E)** Training set. **(B, D, F)** Validation set.

The predictive performance of different models in the training and validation sets is shown in **Figure 3** and **Table 4**. The predictive performance of the combined model was higher than that of other single models. In the training set, the AUC of the combined model was 0.838 (95% CI, 0.799-0.877), 0.855 (95% CI, 0.817-0.894), and 0.906 (95% CI, 0.869-0.943), respectively. In addition, we used an external validation set to verify the accuracy of the combined model, and the AUC was 0.813 (95% CI, 0.740-0.886), 0.852 (95% CI, 0.790-0.913), and 0.875 (95% CI, 0.781-0.929), respectively. In addition, when distinguishing between Del-19 and L858R mutations, the prediction model’s performance constructed using random forest was higher than that of other single models. The AUC of the training and validation sets were 0.881 (95% CI, 0.840-0.921) and 0.871 (95% CI, 0.802-0.941), respectively.

**Table 4:** The prediction performance of different models in the training and validation sets.

Models	AUC	Accuracy	Sensitivity	Specificity	PPV	NPV
**Del-19 mutation *vs.* wild-type**						
**Training set**						
Clinical model	0.719 (0.668-0.770)	0.706 (0.659-0.751)	0.721 (0.589-0.792)	0.697 (0.574-0.743)	0.603 (0.554-0.626)	0.796 (0.763-0.806)
Radiomics model	0.807 (0.765-0.850)	0.747 (0.701-0.789)	0.708 (0.597-0.779)	0.772 (0.651-0.834)	0.665 (0.626-0.686)	0.805 (0.777-0.817)
Combined model	0.838 (0.799-0.877)	0.775 (0.30-0.815)	0.682 (0.565-0.760)	0.834 (0.705-0.896)	0.724 (0.685-0.745)	0.804 (0.776-0.815)
**Validation set**						
Clinical model	0.693 (0.606-0.781)	0.667 (0.581-0.745)	0.648 (0.268-0.787)	0.679 (0.512-0.762)	0.565 (0.349-0.612)	0.750 (0.694-0.771)
Radiomics model	0.779 (0.694-0.864)	0.732 (0.650-0.804)	0.685 (0.592-0.815)	0.762 (0.571-0.952)	0.649 (0.615-0.687)	0.790 (0.738-0.825)
Combined model	0.813 (0.740-0.886)	0.768 (0.689-0.836)	0.648 (0.407-0.759)	0.845 (0.678-0.929)	0.729 (0.629-0.759)	0.789 (0.750-0.804)
**L858R mutation *vs.* wild-type**						
**Training set**						
Clinical model	0.701 (0.651-0.750)	0.679 (0.630-0.725)	0.634 (0.507-0.722)	0.705 (0.600-0.769)	0.564 (0.509-0.596)	0.762 (0.732-0.778)
Radiomics model	0.825 (0.783-0.868)	0.764 (0.719-0.806)	0.772 (0.662-0.842)	0.759 (0.647-0.826)	0.659 (0.623-0.678)	0.847 (0.825-0.858)
Combined model	0.855 (0.817-0.894)	0.756 (0.710-0.798)	0.890 (0.793-0.945)	0.676 (0.531-0.747)	0.623 (0.596-0.637)	0.911 (0.889-0.918)
**Validation set**						
Clinical model	0.697 (0.614-0.781)	0.672 (0.585-0.750)	0.660 (0.458-0.820)	0.679 (0.552-0.798)	0.550 (0.459-0.603)	0.770 (0.732-0.798)
Radiomics model	0.812 (0.737-0.887)	0.746 (0.664-0.817)	0.760 (0.580-0.880)	0.738 (0.547-0.845)	0.633 (0.568-0.667)	0.838 (0.793-0.855)
Combined model	0.852 (0.790-0.913)	0.739 (0.656-0.811)	0.920 (0.740-1.000)	0.631 (0.500-0.774)	0.597 (0.544-0.617)	0.930 (0.913-0.942)
**Del-19 mutation *vs.* L858R mutation**						
**Training set**						
Logistic model	0.581 (0.510-0.651)	0.587 (0.524-0.649)	0.789 (0.593-0.854)	0.395 (0.256-0.473)	0.554 (0.483-0.574)	0.662 (0.559-0.701)
RF model	0.881 (0.840-0.921)	0.786 (0.730-0.835)	0.715 (0.621-0.825)	0.853 (0.769-0.948)	0.822 (0.801-0.842)	0.759 (0.739-0.778)
SVM model	0.601 (0.531-0.671)	0.591 (0.528-0.653)	0.805 (0.634-0.870)	0.388 (0.240-0.465)	0.556 (0.497-0.575)	0.676 (0.564-0.714)
Clinical model	0.660 (0.593-0.726)	0.599 (0.536-0.660)	0.797 (0.645-0.872)	0.411 (0.278-0.506)	0.563 (0.511-0.585)	0.679 (0.589-0.723)
Combined model†	0.906 (0.869-0.943)	0.833 (0.781-0.877)	0.821 (0.738-0.916)	0.845 (0.758-0.923)	0.835 (0.819-0.849)	0.832 (0.816-0.844)
**Validation set**						
Logistic model	0.673 (0.569-0.777)	0.679 (0.582-0.767)	0.827 (0.481-0.924)	0.537 (0.315-0.686)	0.632 (0.500-0.658)	0.763 (0.654-0.804)
RF model	0.871 (0.802-0.941)	0.849 (0.766-0.911)	0.788 (0.394-0.904)	0.907 (0.654-0.981)	0.891 (0.804-0.904)	0.817 (0.763-0.828)
SVM model	0.652 (0.547-0.758)	0.651 (0.552-0.741)	0.808 (0.538-0.962)	0.500 (0.370-0.648)	0.609 (0.509-0.649)	0.730 (0.667-0.778)
Clinical model	0.514 (0.402-0.625)	0.538 (0.438-0.635)	0.692 (0.378-0.885)	0.389 (0.248-0.546)	0.522 (0.373-0.582)	0.568 (0.455-0.648)
Combined model†	0.875 (0.781-0.929)	0.830 (0.745-0.896)	0.885 (0.490-0.962)	0.778 (0.536-0.889)	0.793 (0.680-0.806)	0.875 (0.828-0.889)

AUC, Area under the curve; Del 19, Exon-19 deletion; L858R, Exon-21 L858R point mutation; NPV, Negative predictive value; PPV, Positive predictive value; RF, Random forest; SVM, Support vector machine; vs., versus.

†Combined model: RF model combined Clinical model.

Delong test showed that there were significant differences in AUC values of the three models in the training set between EGFR Del-19 mutation or L858R mutation and wild-type groups (all *P* < 0.05); However, only the AUC value of combined model and clinical model was significantly different in the validation set (*P* < 0.05), and the AUC values between other models were not statistically significant (*P* > 0.05) (**Figures S7A–D**). There were significant differences in AUC values of the combined model and clinical, SVM or Logistic models in the training and validation sets between EGFR Del-19 mutation and L858R mutation groups (all *P* < 0.05). However, the AUC value between combined model and RF model was not statistically significant in the both sets (*P* > 0.05) (**Figures S7E, F**).

### Clinical Application of the Combined Model

**Figure 4:**
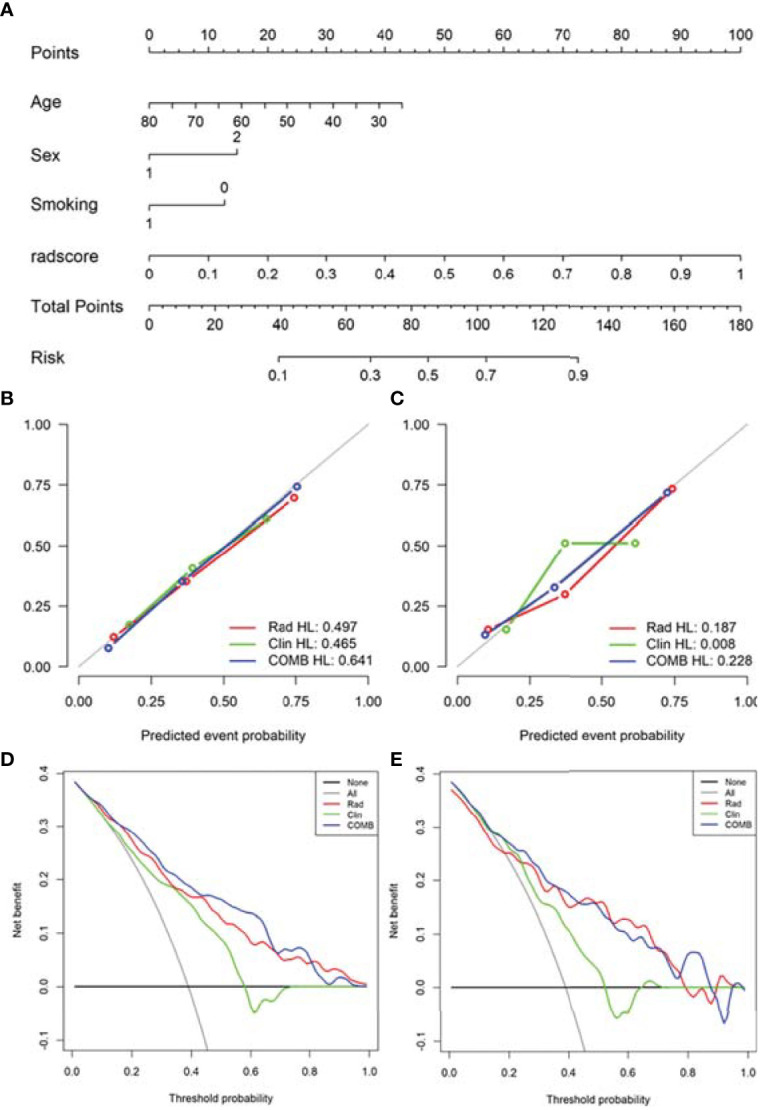
Nomogram was used to identify Del-19 mutation and wild-type. **(A)** Construct a nomogram in the training set based on the combined model. **(B, C)** Calibration curve of the combined model in the training **(B)** and validation **(C)** sets. The x-axis represents the use of the combined model to predict the risk of Del-19 mutation. The y-axis represents the actual Del-19 mutation rate. The green, red, and blue lines represent the distinguishing ability of the clinical, radiomics, and combined models, respectively, while the gray diagonal line represents the ideal evaluation of the ideal model. The closer the fit to the diagonal line indicates the better discrimination ability. **(D, E)** Decision curve analysis for the combined model in the training **(D)** and validation **(E)** sets. The x-axis shows the threshold probability, and the y-axis measures the net benefit. The gray line represents all patients with Del-19 mutation, and the black line represents all patients without Del-19 mutation. The green, red, and blue lines represent the clinical, radiomics, and combined models, respectively.

**Figure 5:**
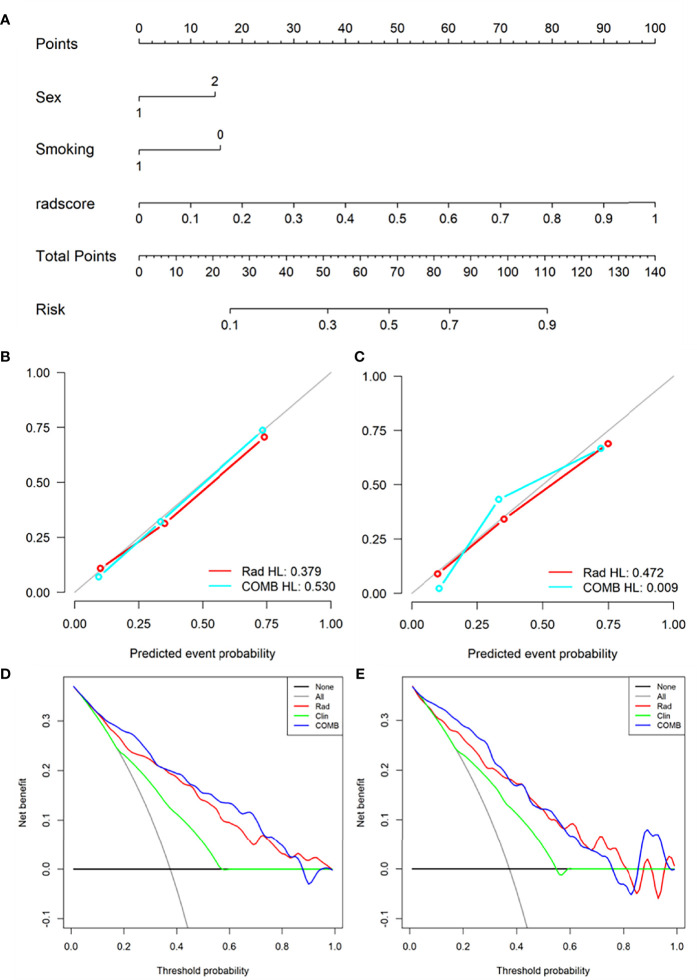
Nomogram was used to identify L858R mutation and wild-type. **(A)** Construct a nomogram in the training set based on the combined model. **(B, C)** Calibration curve of the combined model in the training **(B)** and validation **(C)** sets. **(D, E)** Decision curve analysis for the combined model in the training **(D)** and validation **(E)** sets.

Based on radiomics score and clinical risk factors, we constructed two user-friendly nomograms to predict the mutation status of EGFR molecular subtypes (**Figures 4A** and **5A**). The detailed formula for calculating the radiomics score is shown in the **Supplementary Material** (Result). The calibration curve analysis showed that the probability of Del-19 mutation or L858R mutation predicted by the combined model was highly consistent with the actual possibility, indicating that the model had the best discriminant ability (**Figures 4B, C**, and **5B, C**). Decision curve analysis showed that the combined model threshold in range of 0.18-0.77 have higher net benefit for Del-19 *vs.* wild type and the cutoff value was 0.440 fall in this rang; the combined model threshold in range of 0.16-0.715 have higher net benefit for L858R *vs.* wild type and the cutoff value was 0.389 fall in this range (**Figures 4D, E** and **5D, E**).

**Figure 6:**
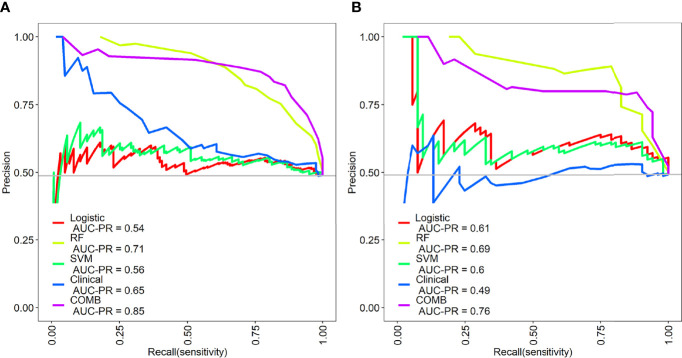
Precision-recall (PR) curves of the different models in the training **(A)** and validation sets **(B)**. PR represents the relationship between precision and recall.The larger the area under the PR curve, the better the model performance.

The precision-recall curves showed that the combined model constructed by the RF model combined with clinical factors had better performance than other single models in predicting Del-19 and L858R mutations (**Figures 6A, B**).

## Discussion

Preoperative noninvasive prediction of EGFR mutant subtypes is a new field that attracts researchers’ attention. It can well overcome some shortcomings of molecular mutation detection based on histology and provide critical information for the rational formulation of targeted therapy in clinical practice. This study established different models based on clinical factors and radiomics features to predict EGFR mutation subtypes. Among them, the combined model showed good predictive performance in the training set. It also had good stability when evaluating the model’s performance in the external validation set, which reveals the feasibility of predicting EGFR molecular subtypes through radiomics features.

Previous studies have found that some clinical variables such as female, non-smokers, patients with histological type of lung adenocarcinoma, and East Asian populations are significantly associated with EGFR mutations (16, 17, 20, 30, 31). Our previous research has also confirmed this (12, 15). However, these studies did not further analyze the correlation between EGFR mutation subtypes and clinical variables. In our study, sex, smoking history, and age were significantly different in the EGFR mutation subtypes group. Compared with EGFR wild-type patients, Del-19 mutation patients were more common in females and non-smokers, and L858R mutation patients were more common in females and non-smokers. Compared with patients with Del-19 mutation, patients with L858R mutation were relatively older. Only patients with Del-19 mutation and L858R mutation were selected because they are the most common mutations in EGFR mutation subtypes. The two mutation sites most related to the sensitivity of EGFR TKI treatment.

Some previous studies have predicted the mutation status of EGFR molecular subtypes based on CT radiomics features and achieved promising results. For example, Li and colleagues (26) retrospectively collected 312 patients with NSCLC, and 580 radiomics features were extracted from each patient’s CT images to construct a model to predict EGFR mutation subtypes (Del 19 and L858R). The test set’s AUC for predicting Del 19 and L858R mutations were 79.3% and 77.5%, respectively. Similarly, Zhao et al. (25) included 637 patients with lung adenocarcinoma in their study to predict EGFR mutation subtypes, and extracted 475 radiomics features to construct a model. The results showed that the AUC in the training and validation datasets were 68.9% and 75.7%, respectively. However, these studies did not distinguish between the Del-19 mutation and the L858R mutation, limiting the clinical applicability of these studies. In this study, we not only distinguished between EGFR Del-19 mutation or L858R mutation and EGFR wild-type. More importantly, we further distinguished the Del-19 mutation and the L858R mutation, and achieved good prediction performance. The training and validation sets’ AUC was 90.6% and 87.5%, respectively. Therefore, our research may be more in line with actual clinical needs.

In this study, whether in the training or validation sets, the combined model established by clinical factors combined with radiomics features can improve the diagnostic performance of identifying EGFR molecular subtypes. Liu et al. (24) included 263 patients with lung adenocarcinoma in their study to detect EGFR mutation status and its molecular subtypes. Among the 6 models established, the combined model had better distinguishing ability than the model that only uses radiomics features or clinical factors. Tu et al. (20) included 404 NSCLC patients in their study to predict EGFR mutation status, and the comprehensive model showed higher predictive performance than any other single model. Jia et al. (32) also showed that a comprehensive model with radiomics features combined with clinical factors had better diagnostic performance than a single model. It shows that adding clinical factors to the radiomics model can improve the diagnostic performance of the model.

In recent years, the study of radiomics in predicting tumor gene mutations has attracted extensive attention from researchers (20, 22, 32, 33). The intrinsic relationship between the radiomics features and EGFR mutation status in patients with lung adenocarcinoma can be further explored through data mining to guide clinical decision-making, predict prognosis and evaluate efficacy (19, 22, 32). This study investigated the relationship between radiomics features and EGFR molecular subtypes. Among these features, most of them were texture features, indicating that texture features were more closely related to EGFR molecular subtypes. The human visual system cannot recognize these features, nor can they be interpreted as specific meanings (34, 35). We observed that radiomics features, including logarithm_glcm_Correlation, wavelet.LLL_glszm_Zone Entropy (ZE), and gradient_glszm_Gray Level Non-Uniformity Normalized (GLNN), etc. were associated significantly with Del-19 mutation. Among them, logarithm_glcm_Correlation and wavelet.LLL_glszm_ZE reflected the image texture of the tumor area, and gradient_glszm_GLNN reflected the gray image value of the tumor area (36). Compared with the EGFR wild-type group, the values of these features were higher in Del-19 mutation, indicating that the image texture and gray image values were related to Del-19 mutation. Lbp.3D.m1_firstorder_10Percentile and Lbp.3D.m1_firstorder_Skewness, etc. were associated significantly with L858R mutation. They reflected the voxel intensity of the image (36). Compared with the EGFR wild-type group, the values of these features were higher in L858R mutation, indicating that the image voxel intensity was related to L858R mutation. Therefore, radiomics features as a new auxiliary tool can predict EGFR molecular subtypes.

Compared with the radiomics model based on only containing radiomics features, incorporating preoperative clinical factors of the nomogram showed the best predictive performance. This user-friendly nomogram will help clinicians easily predict EGFR molecular subtypes in clinical practice. The results were more practical than a single model and can be used for clinical applications in patients with lung adenocarcinoma undergoing CT scans. The task of Del-19 vs. wild-type and L858R vs. wild-type build with linear model (logistic regression) could obtain a satisfactory result, and the linear model is easy for application. Such we didn’t applied nonlinear model. The task of Del-19 vs. L858R was hard, the performance of linear model was not satisfactory, so we add nonlinear model for comparison and selected best model for radiomics score construction.

Our study had several limitations. First, although we collected data from two large medical centers, this was a retrospective study and there may be inevitable selection bias. The conclusions of this study need to be prospectively verified in more centers to improve the clinical applicability of our model. Second, although our study included 728 patients, increasing the sample size will further improve the accuracy of the results of this study. Finally, two radiologists spent a lot of time manually segmenting ROI. Therefore, ROI can be segmented automatically and effectively in future research.

## Conclusion

In conclusion, demonstrated the feasibility of identifying EGFR molecular subtypes through the radiomics features of patients with lung adenocarcinoma, making the formulation of clinically individualized targeted therapy programs more precise and more in line with actual clinical needs, so as to benefit the patients with candidate targeted therapy the most.

## Data Availability Statement

The original contributions presented in the study are included in the article/**Supplementary Material**. Further inquiries can be directed to the corresponding authors.

## Ethics Statement

The studies involving human participants were reviewed and approved by the medical ethics committees of Sichuan Provincial People’s Hospital and Lanzhou University Second Hospital. The ethics committee waived the requirement to participate in written informed consent.

## Author Contributions

GZ, LD, WK, JZ and HP contributed to conception and design of the study. GZ, LD, SL, RQ, SP and XZ organized the database. GZ, YC, JZ and JR performed the statistical analysis. GZ, LD, JZ and YC wrote the first draft of the manuscript. YC, JZ and ZZ wrote sections of the manuscript. All authors contributed to manuscript revision, read, and approved the submitted version.

## Funding

This study received funding from the Sichuan Provincial Cadre Health Research Project (No. Chuan Gan Yan 2022-208), Qinghai Province "Kunlun Talents High-end Innovation and Entrepreneurial Talents" Top Talent Cultivation Project, Medical Science and Technology Research Fund Project of Guangdong Province (B2022144), Science and Technology Plan Fund of Guizhou Provincial (Qiankehe Foundation-ZK [2022] General 634), and Doctoral research start-up fund project of Zunyi Medical University (BS2021-03).

## Conflict of Interest

Author JR was employed by GE Healthcare. Author XZ was employed by Philips (China) Investment Co., Ltd.

The remaining authors declare that the research was conducted in the absence of any commercial or financial relationships that could be construed as a potential conflict of interest.

## Publisher’s Note

All claims expressed in this article are solely those of the authors and do not necessarily represent those of their affiliated organizations, or those of the publisher, the editors and the reviewers. Any product that may be evaluated in this article, or claim that may be made by its manufacturer, is not guaranteed or endorsed by the publisher.
