# Supplementary material for: Development of a Nomogram Based on 3D CT Radiomics Signature to Predict the Mutation Status of EGFR Molecular Subtypes in Lung Adenocarcinoma: A Multicenter Study
Source: Front Oncol. 2022 Apr 29;12:889293. doi: 10.3389/fonc.2022.889293 (PMC9098955; doi:10.3389/fonc.2022.889293)
Supplement: Supplementary file 1 [file DataSheet_1.doc]

**Supplementary Materials**

**Methods**

**Radiomic feature extraction**

In total, 1727 radiomics features were extracted from each volume of interest (VOI) of the CT images. All specific calculation formulas could be easily obtained in the open-source software package PyRadiomics 3.0.1 or previous studies [1]. Here, we only listed several categories that these features could be divided into. Details of radiomics features were as follows:

- 1. 16 shape features,
  2. 324 first order features,
  3. 1387 texture features,

ⅰ. 418 gray-level co-occurrence matrices (GLCM) features,

ⅱ. 304 gray-level run-length matrix (GLRLM) features,

ⅲ. 304 gray-level size zone matrix (GLSZM) features,

ⅳ. 95 neighboring gray tone difference matrix (NGTDM) features,

ⅴ. 266 gray-level dependence matrix (GLDM) features.

First order features and texture features were extracted from original pictures as well as eight filters, including Wavelet filter, Laplacian of Gaussian (LoG) filter, Local Binary Pattern (LBP) 3D filter, Square filter, Square Root filter, Logarithm filter, Gradient filter and Exponential filter. The shape features were extracted from original pictures.

**References**

1. van Griethuysen JJM, Fedorov A, Parmar C, Hosny A, Aucoin N, Narayan V, et al. Computational Radiomics System to Decode the Radiographic Phenotype. Cancer Res. 2017; 77(21): e104-e7.

**Result**

**Radiomics feature selection and model establishment**

The radiomics score (radscore) of radiomics model was the linear combination of model coefficient.

For Del-19 vs. wild model:

radscore=-0.654+0.625×logarithm_glcm_Correlation+0.776×wavelet.LLL_glszm_ZoneEntropy-0.478×wavelet.LLL_gldm_DependenceEntropy-0.733×lbp.3D.k_glrlm_ShortRunEmphasis-0.800×lbp.3D.k_firstorder_10Percentile+0.746×lbp.3D.m2_glrlm_ShortRunLowGrayLevelEmphasis-0.983×lbp.3D.k_ngtdm_Busyness+0.954×lbp.3D.m1_firstorder_Skewness-0.645×wavelet.LLL_glszm_SmallAreaHighGrayLevelEmphasis-1.111×gradient_glszm_GrayLevelNonUniformityNormalized+0.416×wavelet.HHL_firstorder_Maximum-0.866×log.sigma.3.0.mm.3D_glszm_SmallAreaLowGrayLevelEmphasis-0.421×square_glszm_ZoneEntropy+1.483×log.sigma.1.0.mm.3D_glcm_InverseVariance

For L858R vs. wild model: radscore=-0.601-0.890×lbp.3D.m1_firstorder_10Percentile+1.326×lbp.3D.m1_firstorder_Skewness+0.719×lbp.3D.m2_glrlm_ShortRunLowGrayLevelEmphasis-0.349×lbp.3D.k_firstorder_90Percentile+1.449×lbp.3D.k_glrlm_LongRunHighGrayLevelEmphasis-0.481×log.sigma.1.0.mm.3D_firstorder_Range+0.564×log.sigma.1.0.mm.3D_glcm_InverseVariance-0.407×log.sigma.3.0.mm.3D_glrlm_LowGrayLevelRunEmphasis+1.475×square_gldm_DependenceVariance+1.164×squareroot_glszm_GrayLevelNonUniformityNormalized-1.775×wavelet.LLH_ngtdm_Busyness-0.284×wavelet.LHH_glrlm_LongRunHighGrayLevelEmphasis+0.581×wavelet.LLL_glszm_ZoneEntropy

For Del-19 vs. L858R model (RF), the final hyper-parameter with ntree: 50 and mtry: 2.

**Figures**

**Figure S1.** Boxplot showing fourteen radiomics features that were significantly different between Del-19 mutant and wild type groups in the training set.


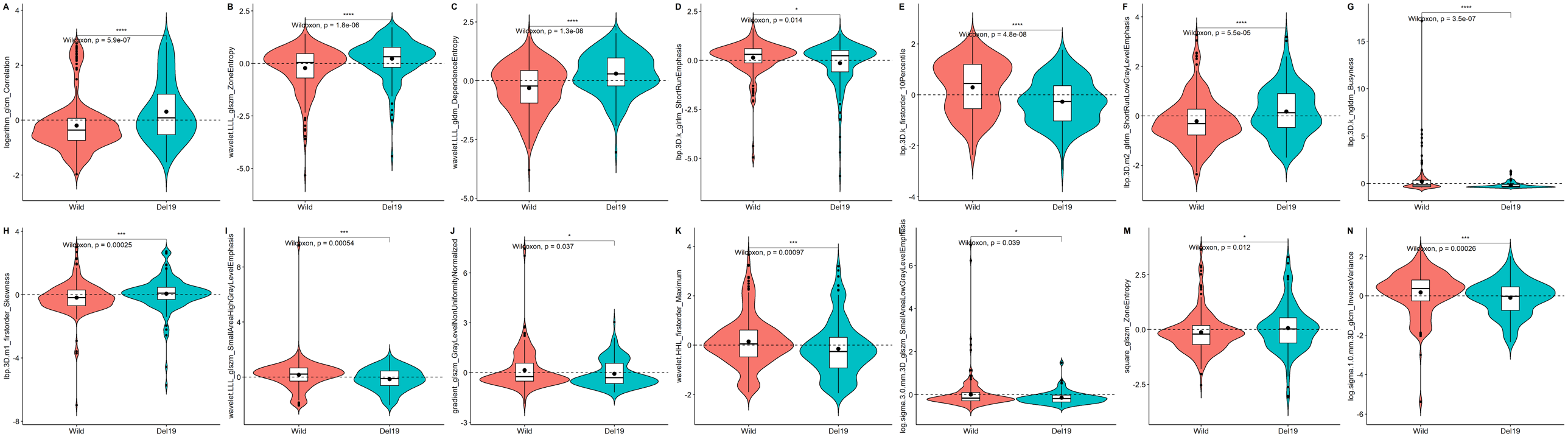


**
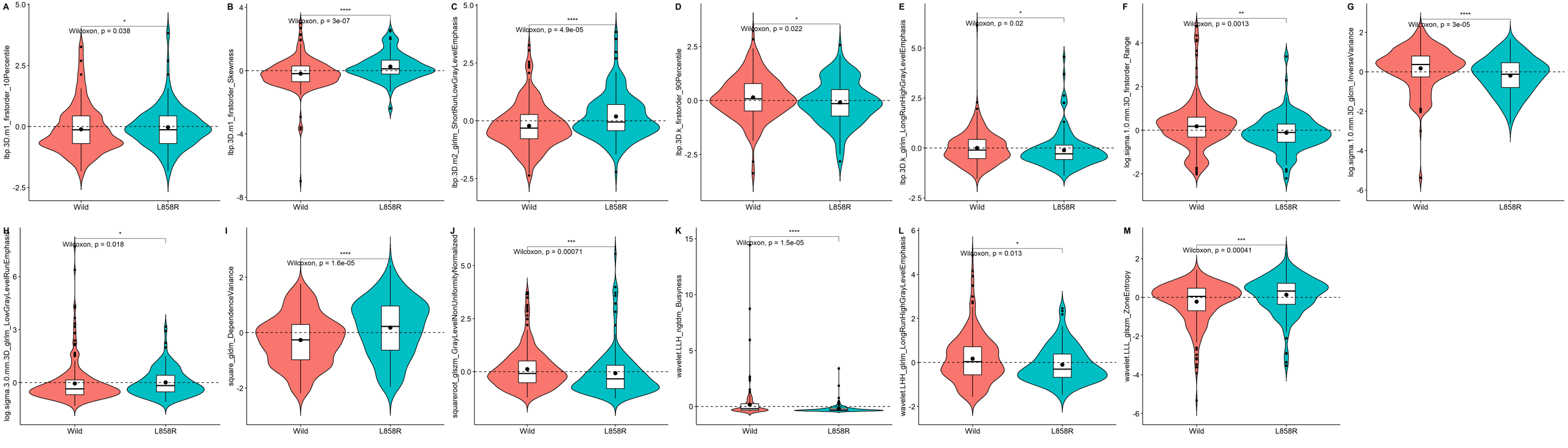
**

**Figure S2.** Boxplot showing thirteen radiomics features that were significantly different between L858R mutant and wild type groups in the training set.

**
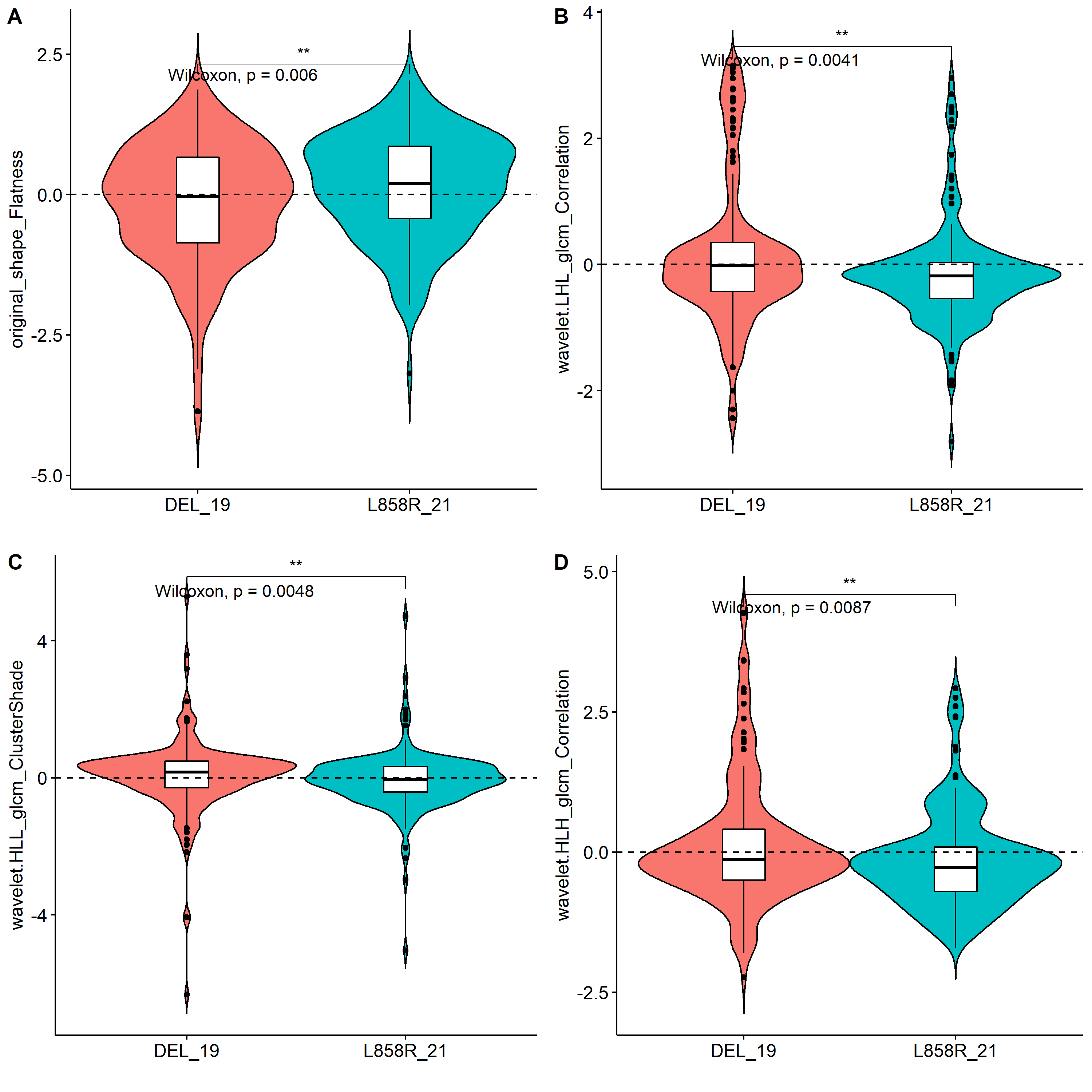
**

**Figure S3**. Boxplot showing four radiomics features that were significantly different between Del-19 mutant and L858R mutant groups in the training set.

**
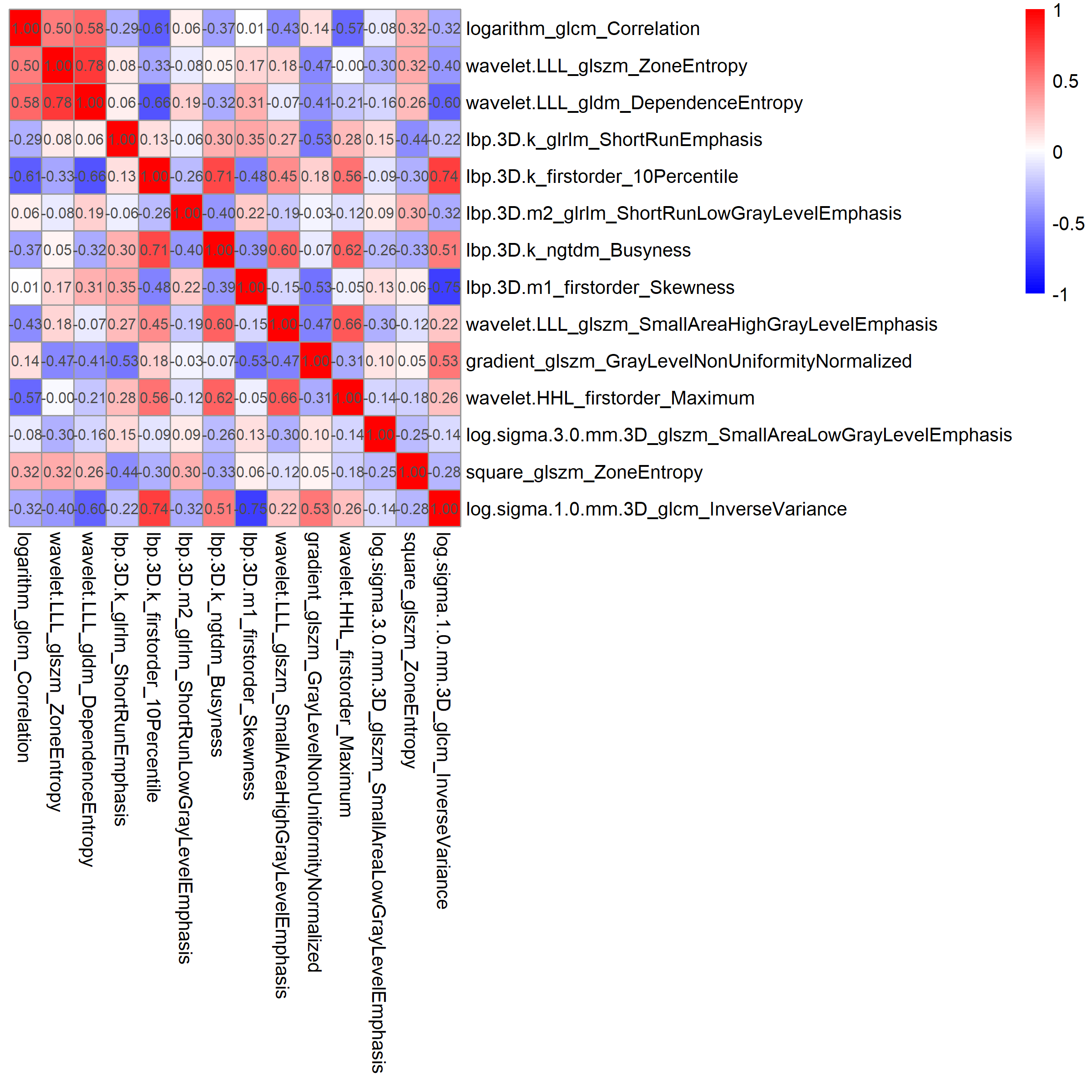
**

A

**
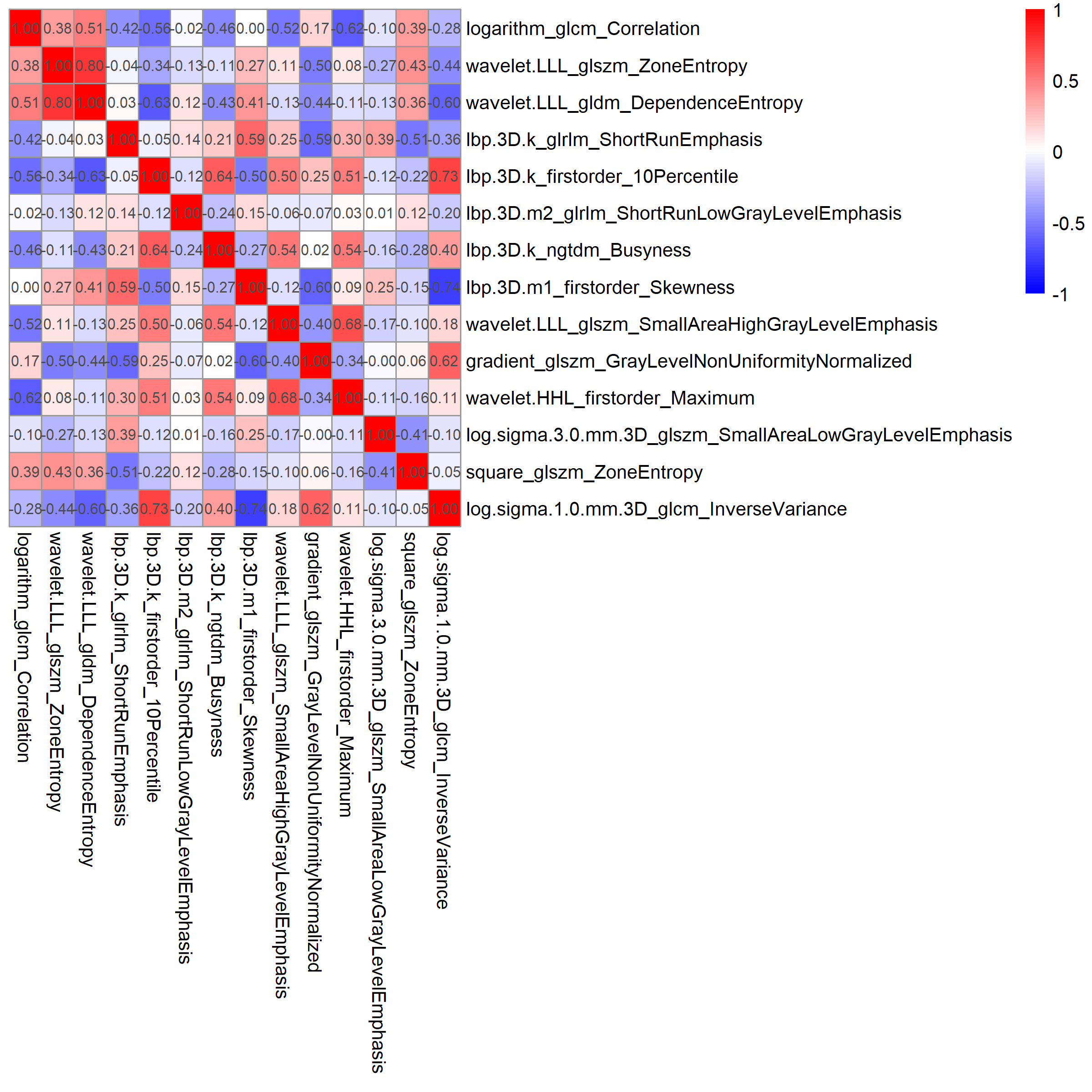
**

B

**Figure S4.** Related heat maps in training (A) and validation (B) sets. Correlation analysis showed that the absolute value of the correlation of each feature between EGFR Del-19 mutation group and wild-type group was less than 0.81.


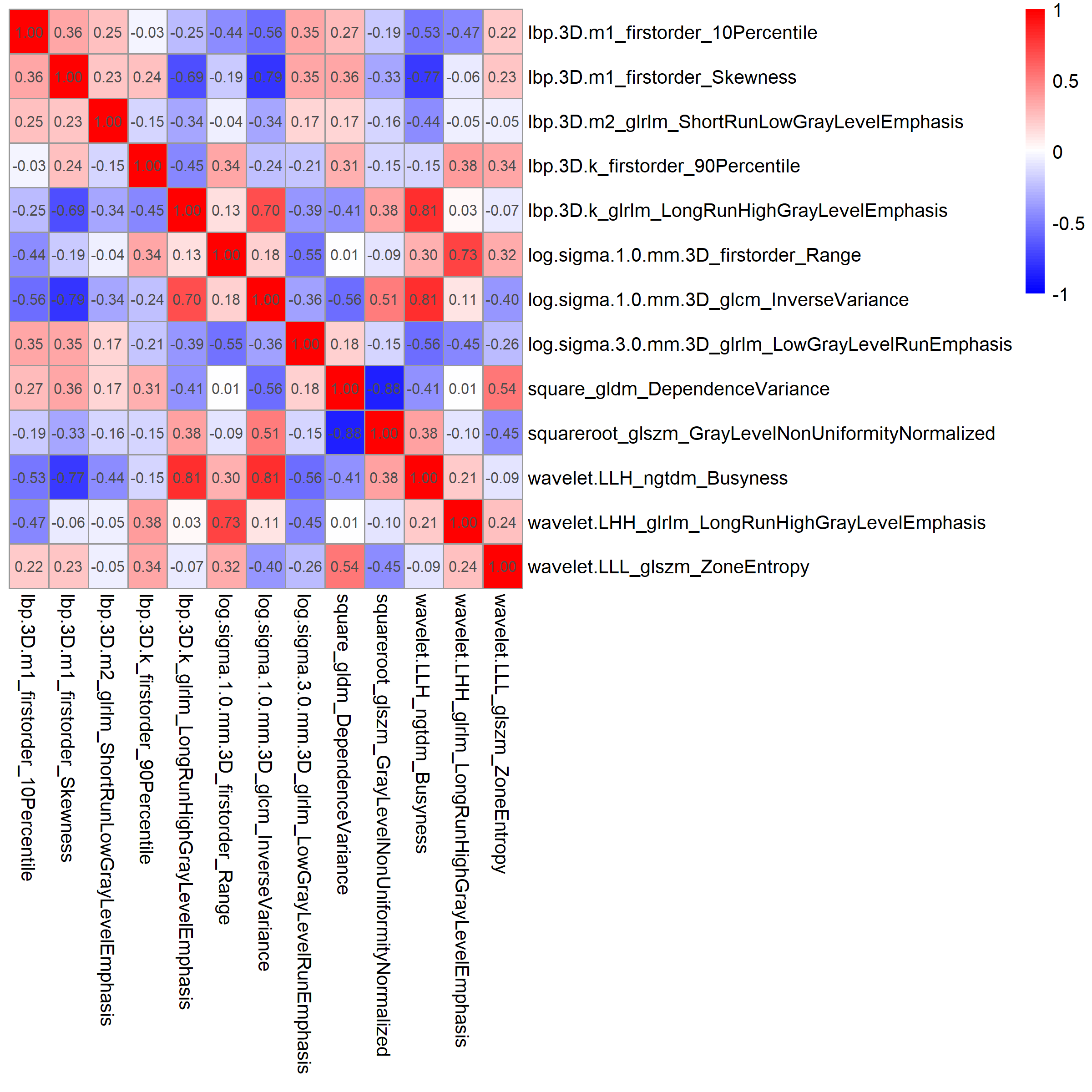


A


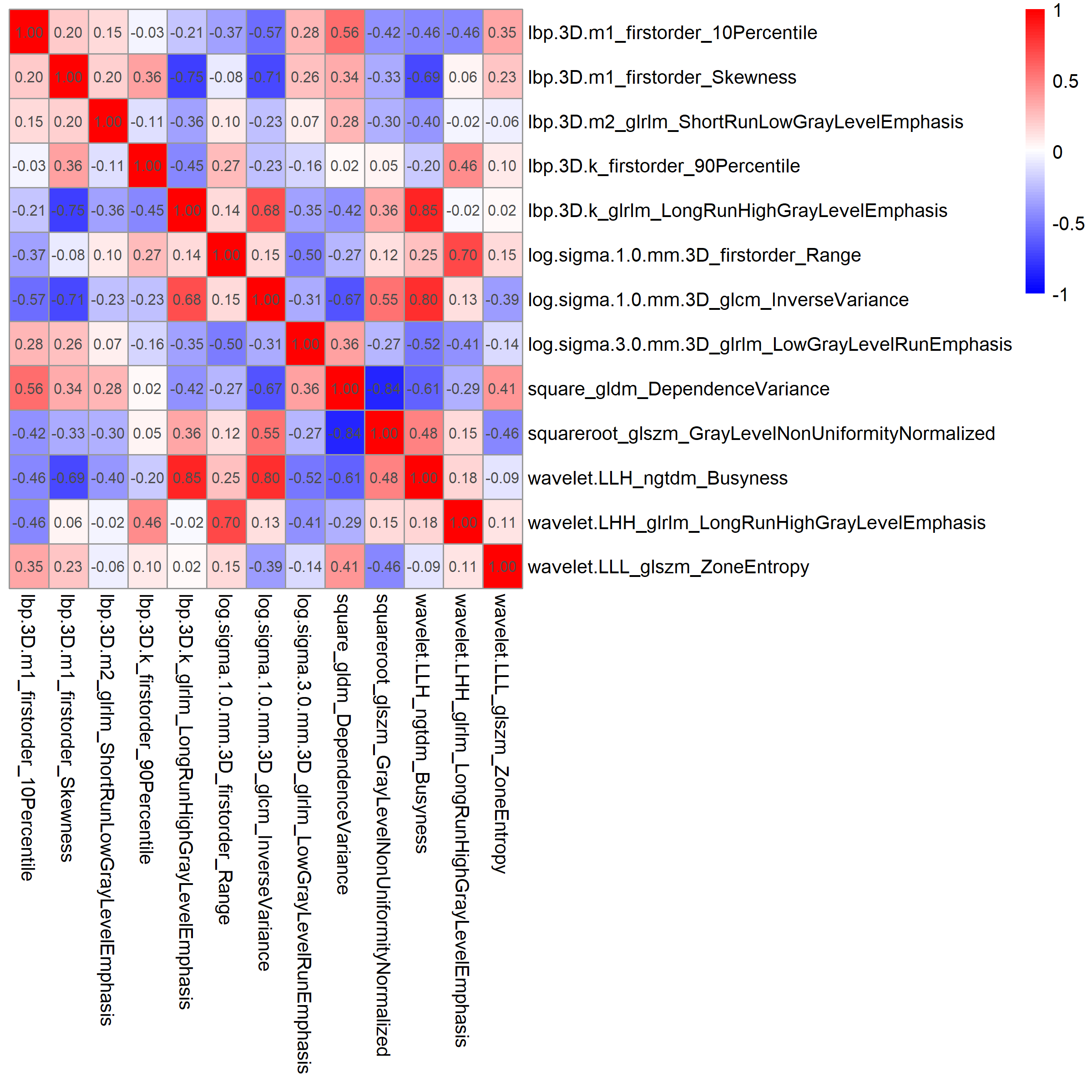


B

**Figure S5.** Related heat maps in training (A) and validation (B) sets. Correlation analysis showed that the absolute value of the correlation of each feature between EGFR L858R mutation group and wild-type group was less than 0.86.

A


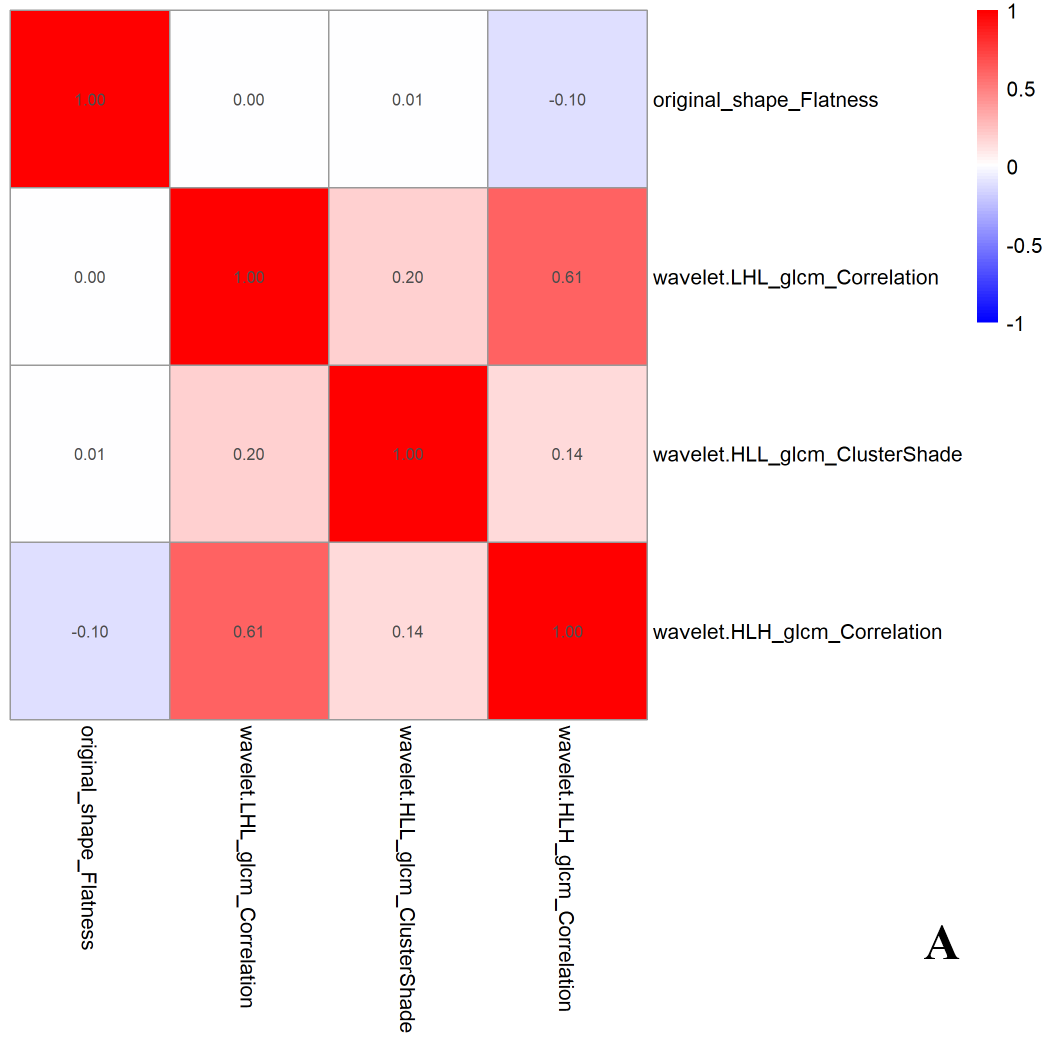


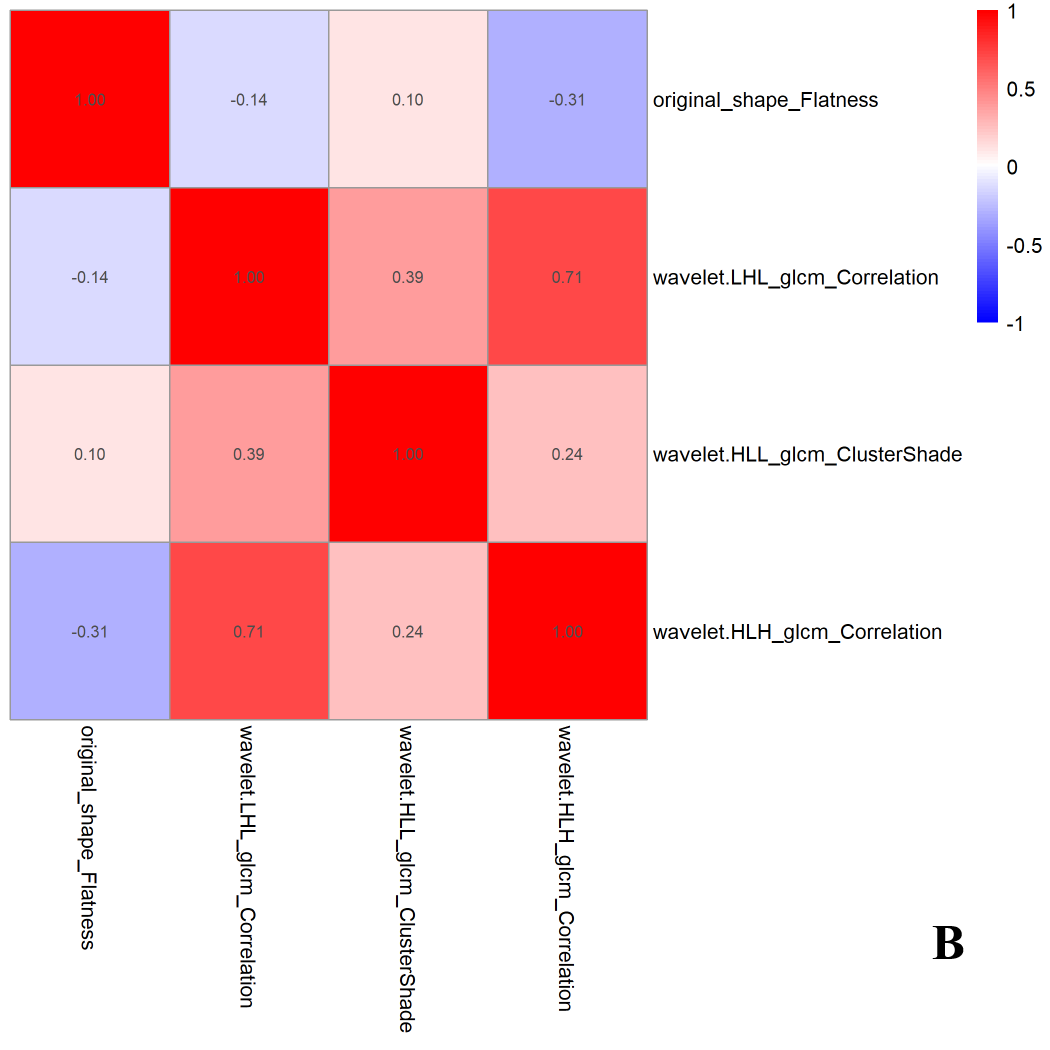


**Figure S6.** Related heat maps in training (A) and validation (B) sets. Correlation analysis showed that the absolute value of the correlation of each feature between EGFR Del-19 mutation group and L858R mutation group was less than 0.72.


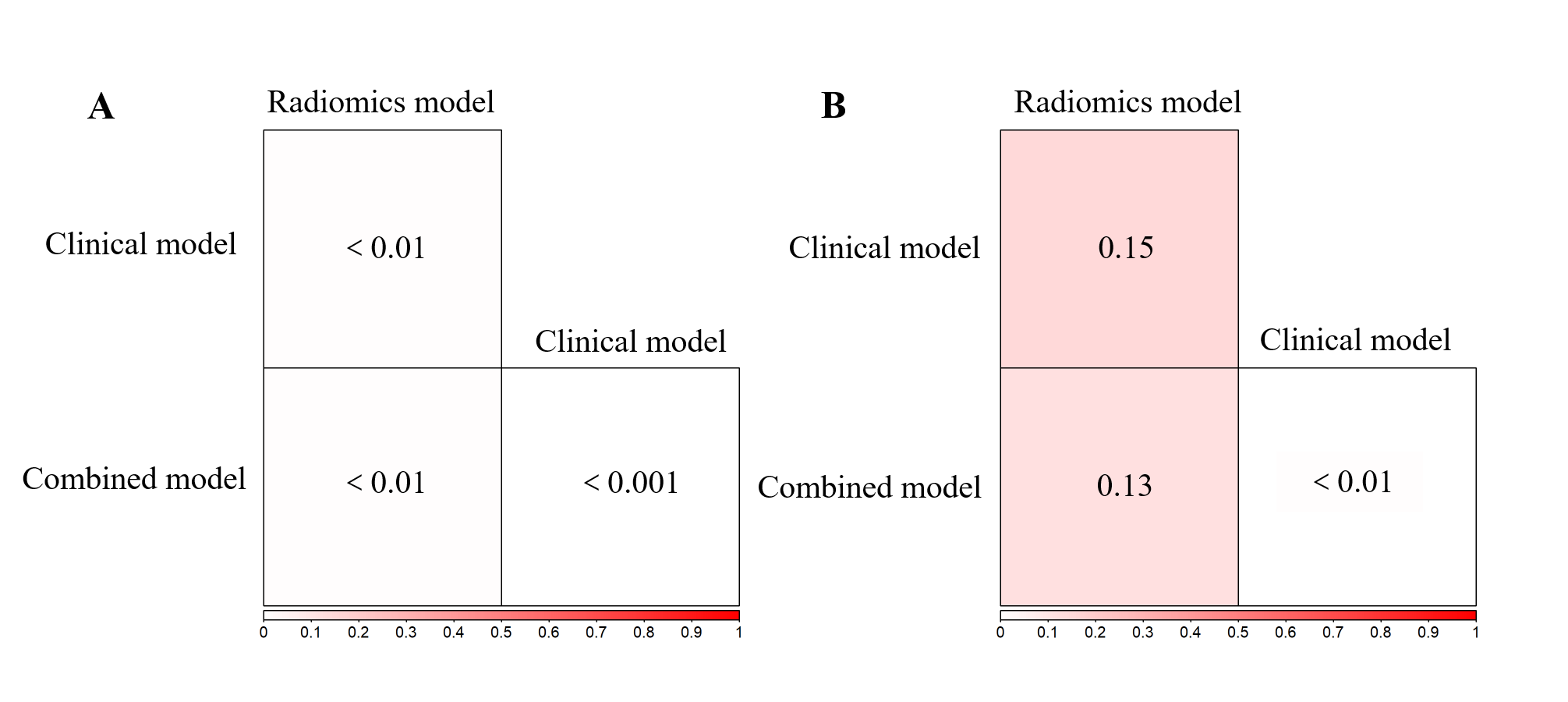


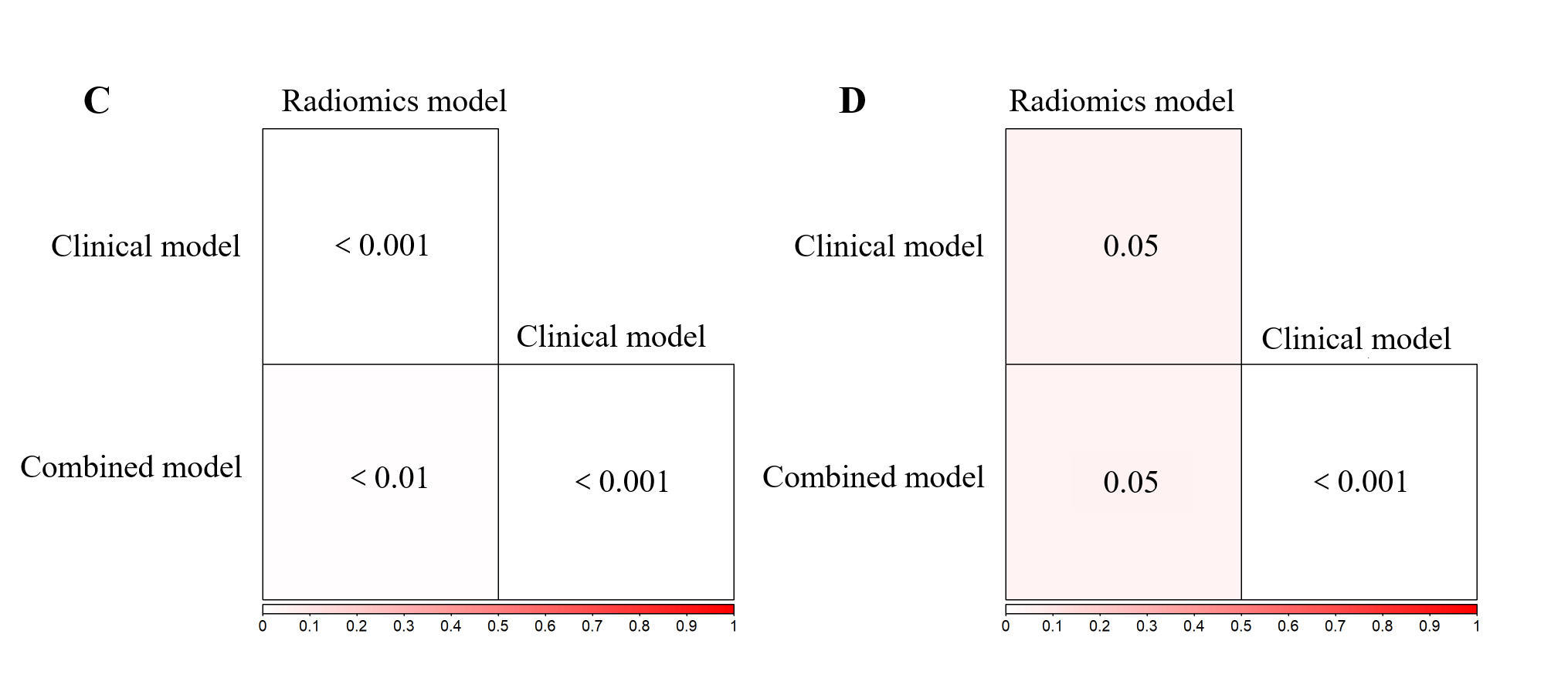


**
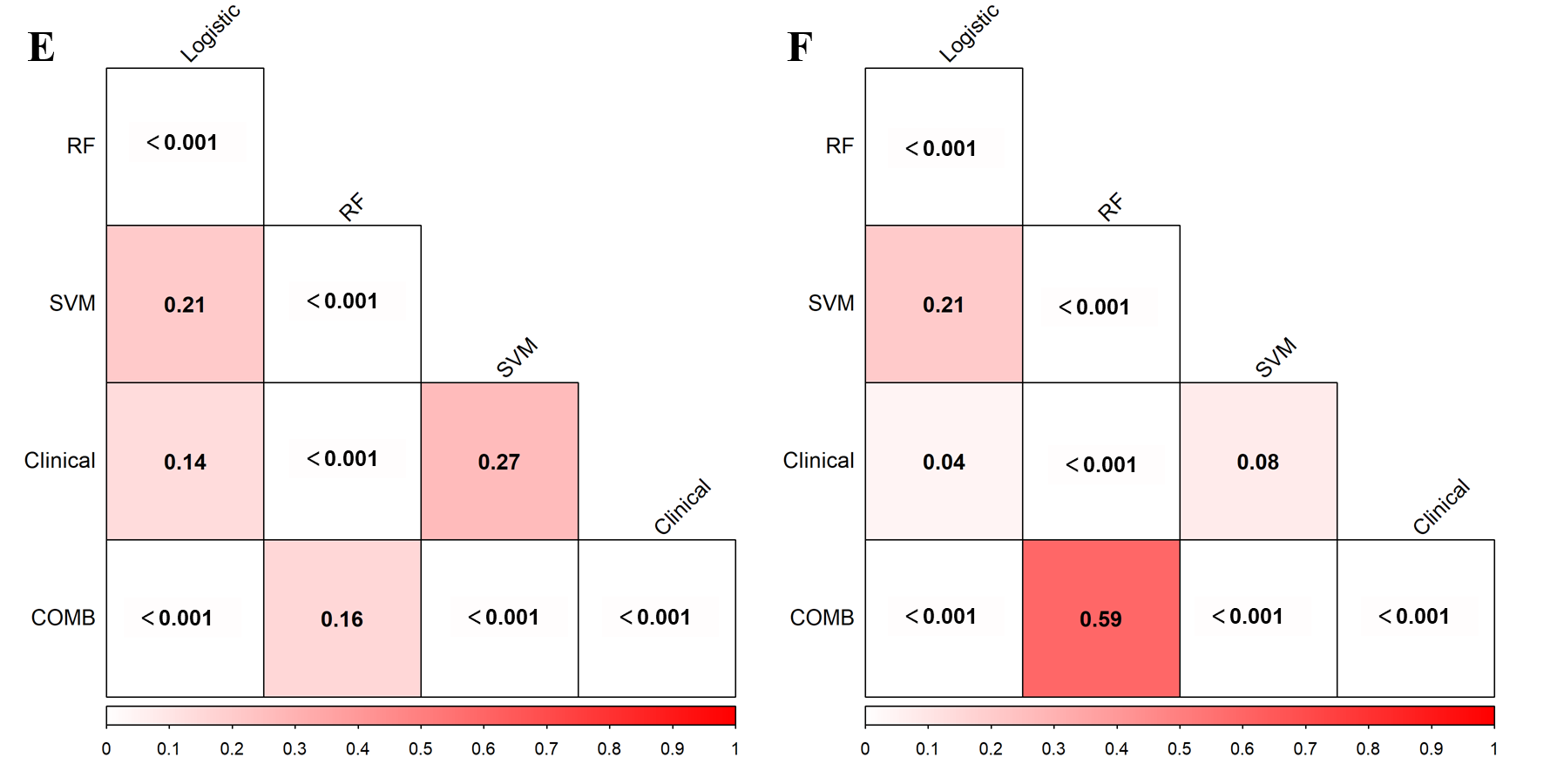
**

**Figure S7.** Delong test was used to compare the performance differences of the three prediction models for predicting the mutant status of EGFR molecular subtypes. (A, B) Del-19 mutation *vs*. wild-type. (C, D) L858R mutation *vs*.wild-type. (E, F) Del-19 mutation *vs*. L858R mutation. (A, C, E) training set. (B, D, F) validation set.

**Tables**

**Table S1. Comparison between EGFR mutation subtype groups (Del-19 *vs*. wild type) and clinical variables in the training and validation sets.**

| Variable | All patients  (N=533) | Training set  (N=395) | Validation set  (N=138) | *P* value |
| --- | --- | --- | --- | --- |
| Age (years) |  |  |  | 0.011 |
| - Mean (SD) | 57.32 (9.18) | 56.70 (9.19) | 59.08 (8.96) |  |
| - Median (Q_1_, Q_3_) | 57.0 (51.0,64.0) | 56.0 (50.0,63.0) | 59.0 (53.0, 65.0) |  |
| - Range | 26.0-79.0 | 26.0-79.0 | 38.0-78.0 |  |
| Sex (%) |  |  |  | 0.791 |
| - Male | 300 (56.3%) | 221 (55.9%) | 79 (57.2%) |  |
| - Female | 233 (43.7%) | 174 (44.1%) | 59 (42.8%) |  |
| Smoking history (%)* |  |  |  | 0.863 |
| - No | 325 (61.0%) | 240 (60.8%) | 85 (61.6%) |  |
| - Yes | 208 (39.0%) | 155 (39.2%) | 53 (38.4%) |  |
| CEA (%) |  |  |  | 0.122 |
| - Normal | 215 (40.3%) | 167 (42.3%) | 48 (34.8%) |  |
| - High | 318 (59.7%) | 228 (57.7%) | 90 (65.2%) |  |
| Lobe location (%)† |  |  |  | 0.294 |
| - Right upper lobe | 192 (36.0%) | 135 (34.2%) | 57 (41.3%) |  |
| - Right middle lobe | 24 (4.5%) | 17 (4.3%) | 7 (5.1%) |  |
| - Right lower lobe | 126 (23.6%) | 97 (24.6%) | 29 (21.0%) |  |
| - Left upper lobe | 100 (18.8%) | 81 (20.5%) | 19 (13.8%) |  |
| - Left lower lobe | 91 (17.1%) | 65 (16.5%) | 26 (18.8%) |  |
| EGFR status (%) |  |  |  | 0.976 |
| - Del-19 | 208 (39.0%) | 154 (39.0%) | 54 (39.1%) |  |
| - Wild type | 325 (61.0%) | 241 (61.0%) | 84 (60.9%) |  |

Note：CEA: Carcinoembryonic antigen; Del-19: Exon-19 deletion mutation; EGFR: Epidermal growth factor receptor; SD: Standard deviation.

* Smoking history was defined as non-smokers (never smoked) and smoking (previously or currently smoked).

† If the tumor crosses the fissure, the lobe location is defined as the lobe in which the tumor predominates.

**Table S2.** Comparison between EGFR mutation subtype groups (L858R *vs*. wild type) and clinical variables in the training and validation sets.

| Variable | All patients  (N=520) | Training set  (N=386) | Validation set  (N=134) | *P* value |
| --- | --- | --- | --- | --- |
| Age (years) |  |  |  | 0.052 |
| - Mean (SD) | 58.60 (9.280) | 58.11 (9.456) | 60.02 (8.628) |  |
| -Median (Q1, Q3) | 58.5 (52.0, 65.0) | 58.0 (52.0, 65.0) | 60.0 (54.0, 66.8) |  |
| - Range | 21.0 - 82.0 | 21.0 - 82.0 | 39.0 - 79.0 |  |
| Sex (%) |  |  |  | 0.608 |
| - Male | 297 (57.1%) | 223 (57.8%) | 74 (55.2%) |  |
| - Female | 223 (42.9%) | 163 (42.2%) | 60 (44.8%) |  |
| Smoking history (%) * |  |  |  | 0.972 |
| - No | 315 (60.6%) | 234 (60.6%) | 81 (60.4%) |  |
| - Yes | 205 (39.4%) | 152 (39.4%) | 53 (39.6%) |  |
| CEA (%) |  |  |  | 0.522 |
| - Normal | 214 (41.2%) | 162 (42.0%) | 52 (38.8%) |  |
| - High | 306 (58.8%) | 224 (58.0%) | 82 (61.2%) |  |
| Lobe location (%)† |  |  |  | 0.105 |
| - Right upper lobe | 179 (34.4%) | 124 (32.1%) | 55 (41.0%) |  |
| - Right middle lobe | 28 (5.4%) | 24 (6.2%) | 4 (3.0%) |  |
| - Right lower lobe | 128 (24.6%) | 99 (25.6%) | 29 (21.6%) |  |
| - Left upper lobe | 104 (20.0%) | 83 (21.5%) | 21 (15.7%) |  |
| - Left lower lobe | 81 (15.6%) | 56 (14.5%) | 25 (18.7%) |  |
| EGFR status (%) |  |  |  | 0.959 |
| - L858R | 195 (37.5%) | 145 (37.6%) | 50 (37.3%) |  |
| - Wild type | 325 (62.5%) | 241 (62.4%) | 84 (62.7%) |  |

Note：CEA: Carcinoembryonic antigen; EGFR: Epidermal growth factor receptor; L858R: Exon 21 L858R point mutation; SD: Standard deviation.

* Smoking history was defined as non-smokers (never smoked) and smoking (previously or currently smoked).

† If the tumor crosses the fissure, the lobe location is defined as the lobe in which the tumor predominates.

**Table S3.** Comparison between EGFR mutation subtype groups (Del-19 *vs*. L858R) and clinical variables in the training and validation sets.

| Variable | All patients  (N=403) | Training set  (N=299) | Validation set  (N=104) | *P* value |
| --- | --- | --- | --- | --- |
| Age(years) |  |  |  | 0.021 |
| - Mean (SD) | 57.16 (8.95) | 56.63 (8.81) | 58.68 (9.22) |  |
| -Median (Q1, Q3) | 56.0 (51.0, 63.0) | 56.0 (50.5, 62.5) | 61.0 (52.5, 65.0) |  |
| - Range | 21.0 - 82.0 | 21.0 - 82.0 | 38.0 - 79.0 |  |
| Sex (%) |  |  |  | 0.618 |
| - Male | 143 (35.5%) | 104 (34.8%) | 39 (37.5%) |  |
| - Female | 260 (64.5%) | 195 (65.2%) | 65 (62.5%) |  |
| Smoking history (%) * |  |  |  | 0.494 |
| - No | 324 (80.4%) | 238 (79.6%) | 86 (82.7%) |  |
| - Yes | 79 (19.6%) | 61 (20.4%) | 18 (17.3%) |  |
| CEA (%) |  |  |  | 0.210 |
| - Normal | 165 (40.9%) | 117 (39.1%) | 48 (46.2%) |  |
| - High | 238 (59.1%) | 182 (60.9%) | 56 (53.8%) |  |
| Lobe location (%)† |  |  |  | 0.211 |
| - Right upper lobe | 141 (35.0%) | 97 (32.4%) | 44 (42.3%) |  |
| - Right middle lobe | 28 (6.9%) | 19 (6.4%) | 9 (8.7%) |  |
| - Right lower lobe | 88 (21.8%) | 72 (24.1%) | 16 (15.4%) |  |
| - Left upper lobe | 88 (21.8%) | 68 (22.7%) | 20 (19.2%) |  |
| - Left lower lobe | 58 (14.4%) | 43 (14.4%) | 15 (14.4%) |  |
| EGFR status (%) |  |  |  | 0.941 |
| - Del-19 | 208 (51.6%) | 154 (51.5%) | 54 (51.9%) |  |
| - L858R | 195 (48.4%) | 145 (48.5%) | 50 (48.1%) |  |

Note：CEA: Carcinoembryonic antigen; Del-19: Exon-19 deletion mutation; EGFR: Epidermal growth factor receptor; L858R: Exon 21 L858R point mutation; SD: Standard deviation.

* Smoking history was defined as non-smokers (never smoked) and smoking (previously or currently smoked).

† If the tumor crosses the fissure, the lobe location is defined as the lobe in which the tumor predominates.

**Table S4.** The final signatures selected from the 3D radiomics features.

| **Radiomics features** | |
| --- | --- |
| **EGFR Del-19 mutation vs. wild-type (fourteen features)** | |
| Radiomic feature | ICC |
| logarithm_glcm_Correlation | 0.850 |
| wavelet.LLL_gldm_DependenceEntropy | 0.871 |
| lbp.3D.k_first-order_10Percentile | 0.909 |
| lbp.3D.k_ngtdm_Busyness | 0.838 |
| wavelet.LLL_glszm_SmallAreaHighGrayLevelEmphasis | 0.921 |
| wavelet.HHL_first-order_Maximum | 0.940 |
| gradient_glszm_GrayLevelNonUniformityNormalized | 0.809 |
| square_glszm_ZoneEntropy | 0.926 |
| wavelet.LLL_glszm_ZoneEntropy | 0.920 |
| lbp.3D.k_glrlm_ShortRunEmphasis | 0.812 |
| lbp.3D.m2_glrlm_ShortRunLowGrayLevelEmphasis | 0.839 |
| lbp.3D.m1_first-order_Skewness | 0.833 |
| log.sigma.3.0.mm.3D_glszm_SmallAreaLowGrayLevelEmphasis | 0.835 |
| log.sigma.1.0.mm.3D_glcm_InverseVariance | 0.931 |
| **EGFR L858R mutation vs. wild-type (thirteen features)** | |
| lbp.3D.m1_firstorder_10Percentile | 0.835 |
| lbp.3D.m2_glrlm_ShortRunLowGrayLevelEmphasis | 0.933 |
| lbp.3D.k_glrlm_LongRunHighGrayLevelEmphasis | 0.909 |
| log.sigma.1.0.mm.3D_glcm_InverseVariance | 0.832 |
| square_gldm_DependenceVariance | 0.919 |
| wavelet.LLH_ngtdm_Busyness | 0.907 |
| wavelet.LLL_glszm_ZoneEntropy | 0.825 |
| lbp.3D.m1_firstorder_Skewness | 0.928 |
| lbp.3D.k_firstorder_90Percentile | 0.889 |
| log.sigma.1.0.mm.3D_firstorder | 0.904 |
| 0.log.sigma.3.0.mm.3D_glrlm_LowGrayLevelRunEmphasis | 0.905 |
| squareroot_glszm_GrayLevelNonUniformityNormalized | 0.845 |
| wavelet.LHH_glrlm_LongRunHighGrayLevelEmphasis | 0.944 |
| **EGFR Del-19 mutation vs. L858R mutation (four features)** | |
| original_shape_Flatness | 0.825 |
| wavelet.HLL_glcm_ClusterShade | 0.921 |
| wavelet.LHL_glcm_Correlation | 0.858 |
| wavelet.HLH_glcm_Correlation | 0.849 |
